# Doxorubicin drug delivery using an electrospun nanofiber membrane of chitosan–polycaprolactone with metal–organic framework: Box–Behnken optimization, anticancer treatment, and antimicrobial activity

**DOI:** 10.1039/d5ra07539d

**Published:** 2025-12-10

**Authors:** Fahad T. Alotaibi, Malak A. Alamri, Lina M. Alneghery, Ali M. Alaseem, Mohamed G. El-Desouky, Ashraf A. El-Bindary

**Affiliations:** a Department of Anatomy and Physiology, College of Medicine, Imam Mohammad Ibn Saud Islamic University (IMSIU) Riyadh 13317 Saudi Arabia; b Health Sciences Research Center (HSRC), Deanship of Scientific Research, Imam Mohammad Ibn Saud Islamic University (IMSIU) Riyadh 13317 Saudi Arabia; c Department of Biology, College of Science, Imam Mohammad Ibn Saud Islamic University (IMSIU) Riyadh 11623 Saudi Arabia; d Department of Pharmacology, College of Medicine, Imam Mohammad Ibn Saud Islamic University (IMSIU) Riyadh 13317 Saudi Arabia; e Egyptian Propylene and Polypropylene Company Port Said 42511 Egypt; f Chemistry Department, Faculty of Science, Damietta University Damietta 34517 Egypt abindary@du.edu.eg

## Abstract

Metal–organic frameworks (MOFs) have become significant nanocarriers for drug delivery owing to their remarkable high surface area, adjustable porosity, and functional adaptability. This research focused on developing a multifunctional pH-responsive delivery system by encapsulating doxorubicin (DOX) within a lanthanum-based MOF (La-MOF) and integrating this complex into a biocompatible electrospun nanofiber membrane made of polycaprolactone (PCL) and chitosan (CS). The resulting DOX@La-MOF/CS–PCL nanofiber membrane was created by means of a one-step electrospinning technique and extensively analyzed using XRD, FTIR, XPS, SEM, EDX, and BET techniques to confirm its structural honesty, surface morphology, and chemical makeup. Drug release experiments indicated a dual-responsive behavior, demonstrating much higher DOX release at body temperature (37 °C) and in acidic environment (pH 5.0) that mimic the tumor micro environment. According to kinetic analysis, diffusion and erosion worked together to affect the release mechanism, which is consistent with zero-order, first-order, Korsmeyer–Peppas, as well as Higuchi models. *In vitro* tests exhibited strong anticancer effects against liver (HepG2), breast (MCF-7), and skin (A-431) lines of cancer cells. In addition to significant antioxidant and antimicrobial achievement in contrast to *Staphylococcus aureus*, *Escherichia coli*, as well as *Candida albicans*. Further optimization through a Box–Behnken statistical design improved both drug loading and release efficiency. Overall, these findings high spot the potential of the DOX@La-MOF/CS–PCL nanofiber membrane as a versatile and effective platform for controlled drug delivery, cancer treatment, and various biomedical applications.

## Abstract 2

Metal–organic frameworks (MOFs) have become significant nanocarriers for drug delivery owing to their remarkable high surface area, adjustable porosity, and functional adaptability.

## Graphical abstract

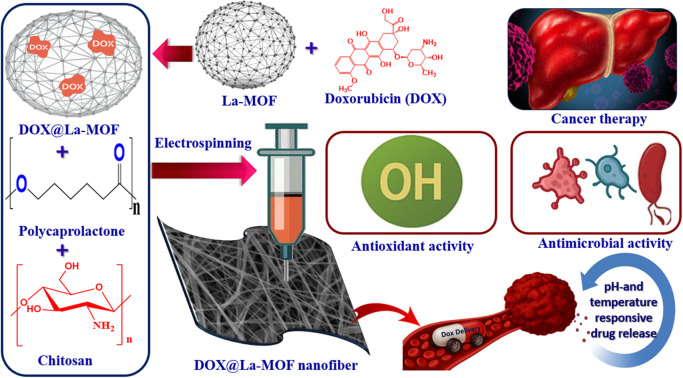

## 1. Introduction

Drug delivery systems (DDS) play a vital role in contemporary medicine as they improve the effectiveness, safety, and adherence of treatments by regulating how drugs are released in terms of rate, timing, and location.^1^ These systems facilitate targeted delivery to specific tissues or areas affected by disease, which helps to reduce side effects while enhancing therapeutic outcomes. They also provide measured and continued drug release, which minimizes the frequency of administration and promotes better compliance among patients.^2–4^ DDS contribute to the stability and bioavailability of drugs that are sensitive or have low solubility, protect active ingredients from early degradation, and aid in overcoming biological challenges like the blood–brain barrier.^5–7^ Furthermore, they lessen systemic toxicity by ensuring that the drug acts locally and support customized medicine by adapting formulations to individual patient needs. In summary, drug delivery systems are vital for maximizing the effectiveness of pharmaceutical products and promoting the development of safe, efficient, and targeted medical therapies.^8^

The use of doxorubicin (DOX) within drug delivery systems is critical because of its recognized effectiveness as a strong chemotherapeutic agent for treating various types of cancer, including breast, ovarian, bladder, and leukemia. Nevertheless, when administered in its unbound form, DOX has several drawbacks, such as non-specific distribution, systemic toxicity (notably cardiotoxicity), rapid elimination from the body, and the risk of developing multidrug resistance in cancer cells.^9^ By incorporating DOX into sophisticated drug delivery systems such as nanoparticles, liposomes, hydrogels, or MOFs its therapeutic effectiveness and safety can be substantially improved. These delivery systems facilitate targeted distribution of DOX to tumor sites, which reduces harm to normal cells and minimizes side effects. Furthermore, these organizations can be designed for controlled and prolonged drug release, allowing for extended circulation time and stable therapeutic concentrations. Functionalized carriers may also react to specific stimuli like pH changes or enzymes, permitting localized release of DOX within the tumor microenvironment. Furthermore, encapsulating DOX can address drug resistance challenges by enhancing cellular uptake and avoiding efflux mechanisms.^10^ In summary, the advancement of DOX-loaded drug delivery systems is essential for optimizing its anticancer capabilities while reducing adverse effects, providing a more effective and patient-centric strategy for cancer treatment.^11^

Metal–organic frameworks (MOFs) are of substantial importance in drug delivery organizations due to their distinct structural and functional characteristics.^12^ These crystalline porous compounds have a very large surface area, tunable pore diameters, and customizable chemical characteristics because they are collected of metal ions otherwise clusters linked by organic linkers. Such attributes position MOFs as excellent carriers for a broad spectrum of drugs, including hydrophobic, hydrophilic, and bio macromolecular substances.^13^ Their significant porosity and expansive surface area facilitate the transport of large quantities of therapeutic agents, and their variable pore dimensions permit size-selective drug loading and release.^14,15^ Moreover, MOFs can be enhanced with stimuli-responsive elements, allowing for regulated or targeted drug release influenced by factors like pH, temperature, enzymes, or light thus promoting site-specific delivery and reducing unintended side effects.^16^ Additionally, when appropriately engineered, MOFs exhibit biocompatibility and biodegradability, making them suitable for *in vivo* use.^17,18^ The adaptability in their design also supports combination therapies, enabling the co-delivery of drugs along with imaging agents or synergistic drug mixtures. In conclusion, the integration of MOFs into drug delivery systems markedly propels the creation of intelligent, effective, and personalized therapeutic platforms characterized by higher drug loading capabilities, controlled release mechanisms, and precise action.^19^

The integration of chitosan (CS) and polycaprolactone (PCL) to create nanofibers through electrospinning, in conjunction with MOFs, establishes a highly actual and multifunctional system for drug delivery.^20^ Chitosan, a natural biopolymer, boasts significant attributes, including excellent biocompatibility, biodegradability, antimicrobial characteristics, and sensitivity to pH changes.^21^ These properties enhance interactions with drugs, promote cellular adhesion, and facilitate controlled release in the acidic environments typical of tumors. PCL contributes additional benefits, including superior mechanical strength, flexibility, and a slower degradation rate, which ensure long-term structural integrity and sustained drug release. When CS and PCL are electrospun together, they form nanofibers characterized by a high surface area to volume ratio and unified porosity, optimizing drug loading and diffusion capabilities. The addition of MOFs to this fibrous structure provides extra advantages owing to their extremely high porosity, adjustable pore sizes, and large surface areas, which enhance drug encapsulation efficiency and protect sensitive therapeutic agents such as doxorubicin.^22^ Furthermore, MOFs can be designed for stimuli-responsive or targeted drug release, promoting precise delivery at the target site while reducing systemic toxicity. This synergistic combination of CS, PCL, and MOFs within electrospun nanofibers results in a robust, biodegradable, and bioactive drug delivery system with improved therapeutic effectiveness, making it particularly suitable for cancer treatment and other controlled-release therapies.^23^

The Box–Behnken Design (BBD) presents several notable benefits for optimizing drug release parameters in pharmaceutical formulations and drug delivery systems. It serves as a response surface methodology (RSM) that systematically and efficiently investigates the relationships between various formulation or process variables and their influence on key outcomes, such as drug release rate, entrapment efficiency, and particle size. One of the primary benefits of BBD is that it needs less experimental runs than full factorial structures, which makes it an economical and time-efficient method that nevertheless provides good statistical power as well as insights into interactions.^24^ BBD is particularly advantageous for addressing nonlinear responses, as it allows for the identification of curvature effects and second-order interactions among variables, which are often present in intricate drug release systems. The design also avoids conducting experiments at extreme low or high levels of all variables simultaneously, thereby minimizing the risks of instability or nonviable formulations. Moreover, BBD supports the creation of predictive mathematical models and response surface plots, which aid in pinpointing optimal formulation conditions. This capability enables researchers to adjust excipient ratios, pH, polymer concentration, or temperature effectively to achieve the desired drug release profile. In summary, employing Box–Behnken design significantly enriches the formulation development process by fostering a comprehensive understanding of factor interactions, enhancing decision-making, and expediting the optimization process within drug delivery research.^25^

This research offerings an pioneering drug delivery organization that responds to pH and temperature by incorporating doxorubicin-loaded lanthanum-based metal–organic frameworks (La-MOFs) into a biocompatible nanofiber matrix made of chitosan and polycaprolactone (CS/PCL), utilizing a one-step electrospinning technique. The resultant DOX@La-MOF nanofiber membrane is comprehensively analyzed and shows improved drug release under conditions that mimic tumors (specifically pH 5.0 and 37 °C). This system stands out from traditional drug delivery methods by integrating structural durability, regulated release in response to dual stimuli, and a wide range of biomedical functions, which encompass anticancer, antimicrobial, and antioxidant properties. Moreover, the request of statistical optimization through the Box–Behnken strategy enhances both the drug loading capacity and release characteristics, thereby provided that a flexible and effective therapeutic strategy. This study marks a notable progress in the creation of intelligent nanocarriers aimed at targeted cancer treatment.

## 2. Experimental

### 2.1. Resources and instruments

The study working reagents categorized as analytical mark, practical in their unrefined state, as indicated in Table S1. A full account of the tools used throughout the research is provided in Table S2.

### 2.2. Manufacture of adsorbent

#### 2.2.1. Manufacture of La-MOF

Utilizing established methods described in other studies, lanthanum metal–organic framework (La-MOF) was produced at room temperature.^26^ Initially, 0.5 g of benzene-1,3,5-tricarboxylic acid (2.38 mmol) was dissolved in 15 mL of a solvent mixture consisting of DMF, EtOH, and H_2_O in equal volumes. Simultaneously, 1.88 g of La(NO_3_)_3_·6H_2_O (4.34 mmol) was liquified in 15 mL of the similar solvent combination (DMF/EtOH/H_2_O). To create a homogenous mixture, these two liquids were then mixed together while being stirred. The entire mixture was then agitated for a full day after 0.5 mL of triethylamine was added. Following this time, the precipitate was filtered out and repeatedly cleaned with 25 mL of DMF. This was then followed by a five-hour drying step at 70 °C.^27^

#### 2.2.2. Procedure for loading drug (DOX@La-MOF)

The successful incorporation of DOX into La-MOFs requires the post-synthesis phase. The La-MOF (100 mg) was mixed with 0.5 mM DOX drug solution (2 mL) and stirred for a period of 48 hours. After this time, the drug-loaded La-MOF (DOX@La-MOF) underwent centrifugation, which was followed by multiple washes with methanol and subsequent drying, preparing it for further experimental applications.^28^ This approach provides notable benefits compared to traditional drug encapsulation methods, as detailed in eqn (1) and (2).1
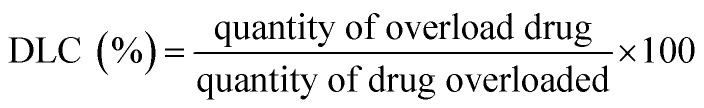
2
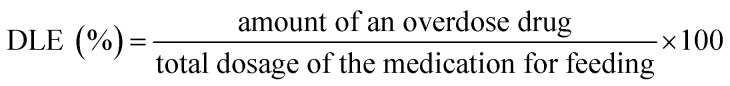


#### 2.2.3. Manufacture of nanofiber membrane (DOX@La-MOF)

**Fig. 1:**
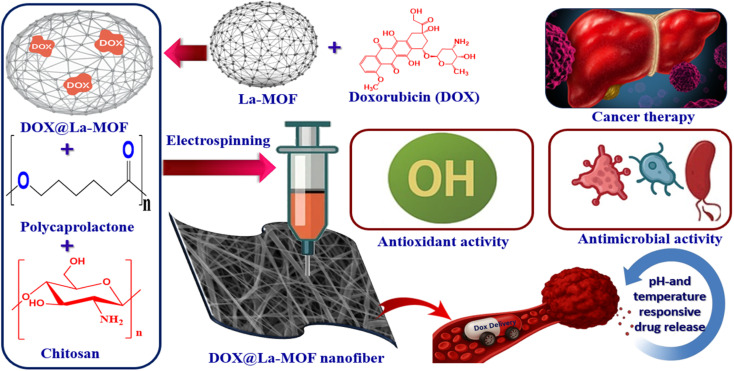
Graphic illustration of the DOX@La-MOF nanofiber membrane production.

Nanofiber supports were synthesized through the electrospinning technique, which is a widely recognized method for producing nanofibers. First, a solution containing 15% (w/v) of polycaprolactone (PCL) dissolved in 90% acetic acid was prepared. This solution was subsequently combined with chitosan (CS), present by 2% (w/v), also in acetic acid (90%), with a volumetric mixing ratio of 7 : 3. Following the preparation of the polymer solution, DOX@La-MOFs were incorporated, and the resultant mixture was agitated overnight to ensure homogeneity. For the ensuing electrospinning procedure, the thoroughly mixed polymer solution was then put into a glass syringe connected to a 20-gauge blunt-tip needle.^29^ The solution was delivered using a syringe pump, which ensured a precise flow degree of 1 mL h^−1^. In order to enable the electrospinning procedure, a high-voltage power supply was employed to generate an electrical potential ranging from 27 to 28.5 kV, applied to the needle. This needle was purposefully placed 15 cm above a collecting plate that was grounded (Fig. 1).

### 2.3. DOX@La-MOF nanofiber membrane drug release

Two phosphate buffer solutions with pH values of 6.2 and 7.4, respectively, and 25 mL of acetate buffer solution with a pH of 5.0 were mixed with 10 mg of DOX@La-MOF nanofiber membrane. Examining the DOX release kinetics from the DOX@La-MOF nanofiber membrane was the aim of this configuration. The release experiments were conducted in controlled settings with constant agitation at 100 rpm at a temperature of 37 °C. The supernatant was obtained using centrifugation techniques after a volume of 1 mL was extracted from the release medium at predetermined intervals. At a wavelength of 480 nm, the released DOX was measured using UV-vis spectrophotometry. The experimental data were compared to a known calibration curve in order to ensure accuracy.^12^ The taster was returned to the particular release mechanism in order to maintain a constant volume. The following equation (eqn (3)) was used to compute the amount of DOX released:3
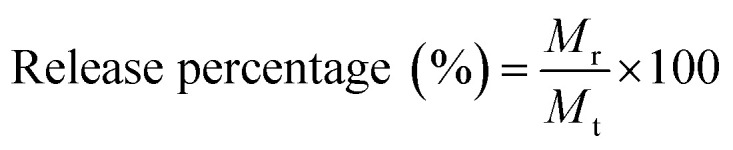


In this case, *M*_t_ represents the total volume of DOX that has been encapsulated, while *M*_r_ represents the quantity of DOX that has been released.

### 2.4. The releases of DOX at different pH levels *via* the DOX@La-MOF nanofiber membrane

The DOX release properties from the DOX@La-MOF nanofiber membrane were assessed using a methodical procedure. This was accomplished by dispersing 10 mg of the nanofiber particles in 20 mL of phosphate buffer solution (7 and 8) along with an acetate buffer solution (pH 5.0). Under carefully regulated settings, the trials were carried out at 37 °C with a shaking frequency of 100 rpm to provide homogeneous mixing. Samples of one mL of the release medium were removed for examination at prearranged intervals.^29^ Centrifugation was used for removing the supernatant for additional study. Using a previously developed calibration curve to ensure accurate quantification, the released concentration of DOX from the membrane was measured by means of UV/Vis spectrophotometry at an excitation wavelength of 480 nm.^12^ The taster was put back into the unique release system to ensure a consistent volume. The formula given in eqn (4) was used to compute the quantity of DOX released:4
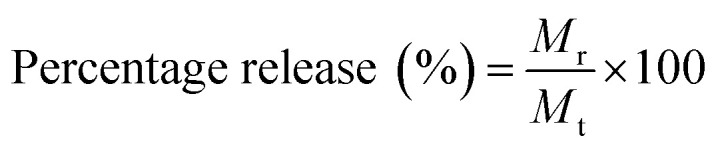


In this model, *M*_r_ signifies the amount of DOX that is still unbound or in a free state, and *M*_t_ represents the total quantity of DOX that has been given to the organization in excess.

### 2.5. Biochemical procedures

#### 2.5.1. Antioxidant activity

The DPPH assay procedure was used to evaluate the radical scavenging capacity of La-MOF, free DOX, and DOX@La-MOF membranes (Table S3). In particular, the 2,2-diphenyl-1-picrylhydrazyl (DPPH) radical was created and then stored in a freshly made methanol solution at a concentration of 0.004 percent w/v in a dark place at a regulated temperature of 10 °C. The test samples were then prepared into a methanol solution. To make the evaluation easier, an aliquot of 40 µL was then added to 3 mL of the DPPH solution.^30^ A UV-visible spectrophotometer was used to measure values of absorbance in real time. Measurements were made every minute until a plateau in absorbance was attained, specifically at the 15 min timestamp, and observations showed a consistent decline in absorbance at 515 nm.^31^ Furthermore, the absorbance values of the reference material, ascorbic acid, and the DPPH radical (control) were examined. Three repeats of each set of experimentations were directed, and the consequences were then averaged for dependability.^32^ Using eqn (5), the percentage inhibition (PI) of the DPPH radical was resolute.5
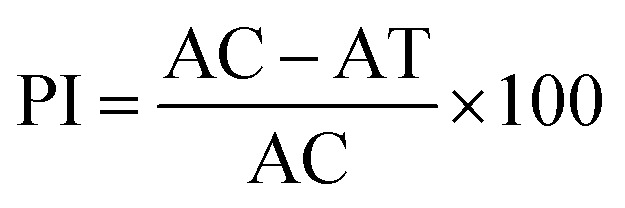


In this scenario, AC serves as the absorbance value recorded for the regulator at the baseline time of *t* = 0 min, whereas AT signifies the absorbance measurement of the sample engaged with DPPH at the time mark of *t* = 16 min.

#### 2.5.2. Cytotoxicity evaluation using the viability assay

The human cancer cell line MCF-7, the human hepatic carcinoma cell line HepG-2, and the skin carcinoma cell line A-431 are the particular cell lines used in this investigation (Tables S4 and S5). To ensure their viability and growth, these cells were maintained in Dulbecco's improved eagle's medium enriched with 50 µg per mL gentamicin, 1% l-glutamine, and 10% heat-inactivated fetal bovine serum.^33,34^ The cells were subculture every two weeks at a temperature of 37 °C in a regulated, humidified situation with 5% CO_2_.^35^

Using 100 µL of the suitable growth medium, cells were first plated in a 96-well format for the cytotoxicity assay. Renewed medium enriched with various dilutions of the test chemical was added after a 24 h incubation period. The chemical compound in question was subjected to a series of two-fold dilutions, which were meticulously dispensed onto the confluent cell monolayers with the aid of a multichannel pipette, ensuring precise application in the flat-bottomed microtiter plates.^34,36^ The plates underwent a further incubation period of 24 h at 37 °C under a controlled moistened air comprising 5% CO_2_. For each concentration class, the test material was divided among three wells as part of the experimental design. The test medication had no effect on the control cells, which were kept apart and didn't need dimethyl sulfoxide. Crucially, the study's findings were unaffected by the attendance of dimethyl sulfoxide in the wells at a maximum level of 0.1 percent. Cell viability was evaluated using a colorimetric test after the 24 h incubation at 37 °C.^37^

In conclusion, upon reaching the end of the incubation phase, the fluids present in each well were extracted. Following this, 1% solution of crystal-violet was applied for a minimum length of 30 min. After the staining time was over, the plates were rinsed with bidistilled water to eliminate any remaining discoloration and wash away any residual stain. To ensure proper interaction, 30% glacial acetic acid was then added to each well and thoroughly mixed. Finally, the absorbance of the plates at a wavelength of 490 nm was measured using a microplate reader. As a background control, the absorbance measurements from wells that were not further stained were used to standardize the experimental results. The cellular baseline, which lacked the investigated chemicals, was used to compare the treated samples. Every experiment was carried out twice to increase the validity of the results. Each compound's cytotoxic effects on the cells were evaluated quantitatively. Using a microplate reader to quantify the optical density, cell viability was determined (eqn (6)).^38^6
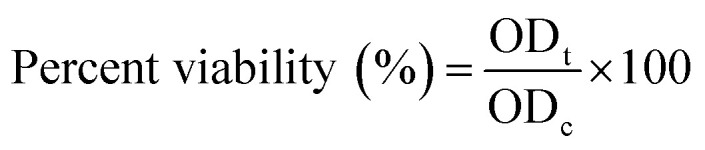


The standard optical density observed in wells subjected to the specific sample is referred to as OD_t_, while the mean optical density of the control cells, which remain unaltered, is indicated as OD_c_. To ascertain the mortality curves for every type of tumor cell following treatment with the specified chemical, a graphic representation of the correlation between the number of surviving cells and the quantity of the medication administered is created. The 50% inhibitory concentration (IC_50_), or the concentration required to have negative impacts on 50% of healthy cells, was calculated using GraphPad Prism software. The visual cues obtained from the dose–response curves associated with each concentration level are used in this computation.^35^

#### 2.5.3. Antibacterial studies

In this work, newly created medium was used to cultivate Gram-negative bacteria, specifically *Escherichia coli*. At the same time, the antibacterial efficiency of La-MOF membranes was evaluated using Gram-positive bacteria, specifically *Staphylococcus aureus*. Comparisons with free DOX and DOX@La-MOF membranes have been included in this assessment.^39^ The agar-well diffusion process was used for the assessment. First, each sterile Petri dish was filled with 40 mL of agar media containing the designated bacterial strains. After that, the plates were let to rest at room temperature until the medium could harden.^40^ A well with a diameter of 6 mm was made by means of a sterile cork borer. Each well of the constructed culture plates limited 100 µL of La-MOF, free DOX, and DOX@La-MOF membrane at a meditation of 10 mg mL^−1^. The plates were then hatched for 24 h at 37 °C. To assess the samples' antimicrobial effectiveness, the widths of the resultant inhibition zones were measured after the incubation. These inhibitory zones, which target both Gram-positive as well as Gram-negative bacterial strains, were then contrasted with those produced by the traditional antibiotic gentamicin.

### 2.6. pH-based medication delivery system

The use of nano-biomaterials that display sensitivity to differences in the pH levels present in the tumor's nearby environment is a well-considered method for the exact delivery of medications to tumor areas.^41^ By taking advantage of the pH differences between surrounding healthy tissues as well as tumor microenvironments, a perfect pH-responsive drug delivery system is created. The development of therapeutic medicines with enhanced sensitivity is made possible by this difference in pH gradients. Nanomaterials in these settings may undergo structural changes as a result of pH shifts from neutral to acidic. Notably, swelling behaviors that improve the release of active therapeutic substances can occur in pharmaceutical delivery systems that use La-MOF nanofiber membrane.^39^

### 2.7. Experimental design

In situations where there are several alternative outcomes, Response Surface Methodology (RSM) serves as an organized method for creating thorough statistical models. The primary objective of RSM is to elucidate the connections between the response parameter and various experimental settings. Additionally, using these models helps to improve the processes that are being studied. Each sample's DOX release efficiency was measured by averaging the outcomes of three separate experimental attempts. The RSM is a methodical technique for creating a variety of planned experiments with the goal of maximizing the essential features of a specific process. In the broader operational environment, this process helps identify the best option. Within this paradigm, Central Composite Design (CCD) is a often used technique for procedural parameter optimization. With a focus on evaluating the efficacy of the DOX@La-MOF membrane, eqn (1) measures the percentage of DOX released from the adsorbent across numerous time intervals (*t*). Additionally, eqn (1) provides a methodical way to estimate the DOX release process's efficacy in percentage terms. Three important factors “adsorbent weight”, “interaction duration”, and “solution pH” have a big influence on the process. The information in Table S6 shows that these elements have been identified because of their unfavorable effects on the adsorption efficacy.^42^

Table S6 gives an organized overview of the highest values connected to each analyzed parameter, effectively condensing the extensive analysis accessible in Table 1 of the Design Expert Software. The purpose of Table 1 is to show the numerous parameter adjustments and their conforming results. Determining the model variables, setting up the experimental setup, and projecting the model's marks are the three crucial phases of the central composite design technique. Every one of these stages requires a careful and comprehensive examination of the outcomes.^43^ After the aforementioned activities are finished, a methodical experimentation model has been created to evaluate the purpose's performance in relative to numerous combinations of input variables. Eqn (7) shows how this has led to the development of a quadratic regression model.^44^

7 *Y* = *β*_0_ + ∑*β*_*i*_*X*_*i*_ + ∑*β*_*ii*_*X*_*i*_^2^ +∑∑*β*_*ij*_*X*_*i*_*X*_*j*_

**Table 1:** The adsorption capacity of DOX release (%) *via* Box–Behnken design was assessed analytically using response surface approach

Run	Real variables			DOX release (%)		
	Temp. (°C)	Time (min)	pH	Experimental	Expected	Residue
1	42	100	6.2	49.95	47.65	2.30
2	33.5	5	7.4	0.344	−2.22	2.57
3	33.5	52.5	6.2	53.7931	53.79	0.0000
4	25	52.5	5	78	74.05	3.95
5	33.5	100	7.4	49.17	47.52	1.65
6	42	52.5	5	48.75	48.48	0.2694
7	33.5	5	5	0.5106	2.16	−1.65
8	33.5	52.5	6.2	53.7931	53.79	0.0000
9	42	52.5	7.4	37.142	41.09	−3.95
10	33.5	100	5	73	75.57	−2.57
11	25	100	6.2	75.92	77.30	−1.38
12	42	5	6.2	0.349	−1.03	1.38
13	33.5	52.5	6.2	53.7931	53.79	0.0000
14	25	5	6.2	0.53097	2.83	−2.30
15	33.5	52.5	6.2	53.7931	53.79	0.0000
16	25	52.5	7.4	48.75	49.02	−0.2694
17	33.5	52.5	6.2	53.7931	53.79	0.0000

The speed coefficient is signified by “*j*” and the resistance constant by “*i*” in this analytical framework. Variables related to resistance, interaction, speed, as well as the constant term are denoted by the constants *β*_0_, *β*_*i*_, *β*_*ii*_, and *β*_*ij*_. Performance indicators like *R*^2^ and *R*_Pred_^2^ were used to evaluate the effectiveness of the projected polynomial classical equation. A higher *R*^2^ value specifies better predictive precision for the model and suggests a stronger association among the model and the observed empirical data.

## 3. Results and discussion

### 3.1. Characterization of DOX@La-MOF nanofiber membrane

#### 3.1.1. X-ray diffraction

**Fig. 2:**
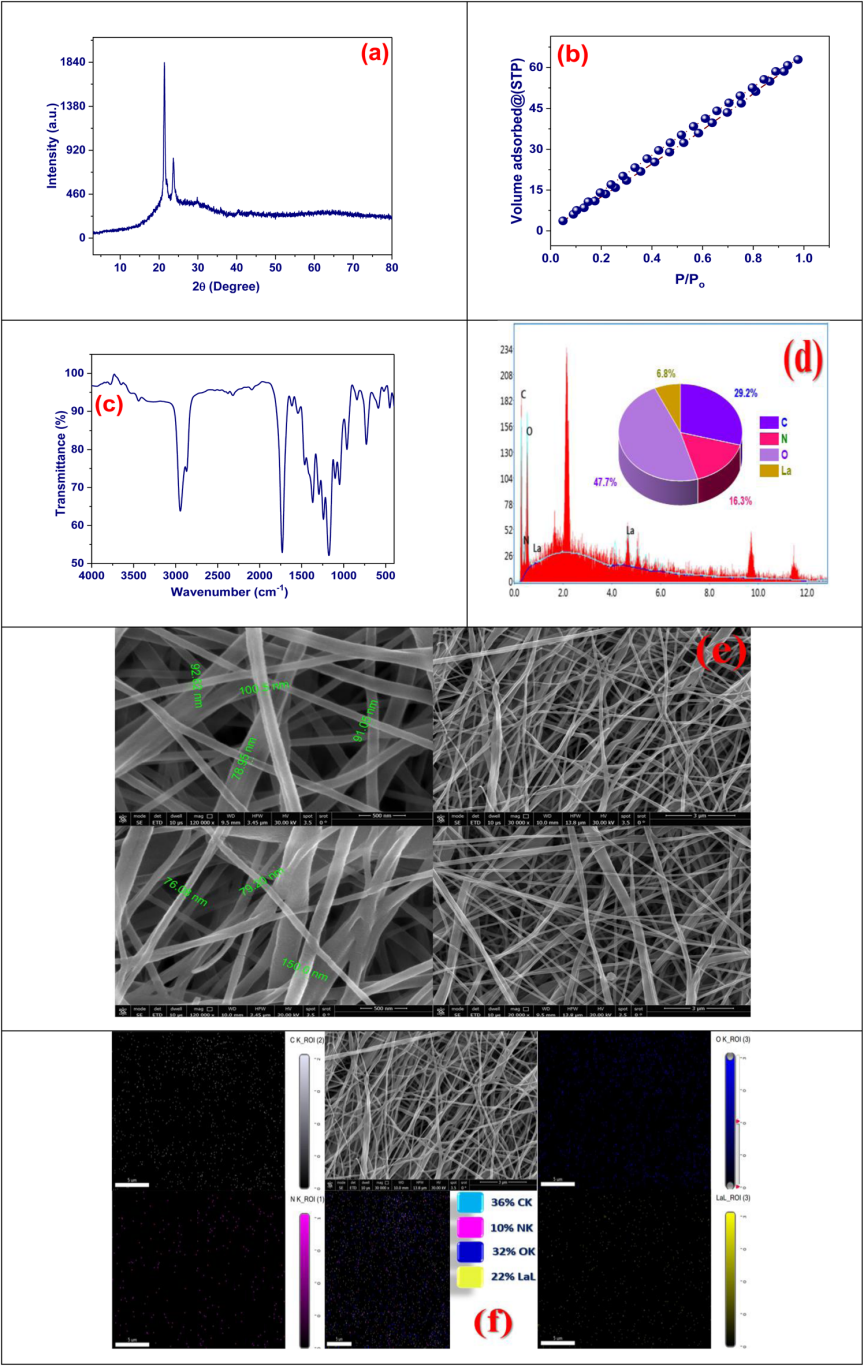
(a) XRD pattern of DOX@La-MOF membrane, (b) N_2_ adsorption/desorption isotherm of DOX@La-MOF, (c) FT-IR of DOX@La-MOF, (d) EDX analysis of DOX@La-MOF, (e) SEM image at different scale, and (f) SEM mapping of DOX@La-MOF.

The X-ray diffraction (XRD) pattern of the DOX@La-MOF nanofiber membrane, illustrated in Fig. 2(a), exposes a composite structure that is both crystalline and amorphous. This outcome can be attributed to the effective electrospinning process employed to integrate the La-MOF into a polymer matrix collected of chitosan (CS) and polycaprolactone (PCL). Notably, the presence of distinct and intense diffraction peaks in the 10–35° 2*θ* range, especially the significant peak observed around 27°, underscores the preservation of La-MOF's crystalline integrity despite the processes of encapsulation and drug incorporation. The identified peaks correspond to the well-organized lattice planes characteristic of the MOF, which suggests that the structural honesty of the La-MOF was preserved during the fabrication procedure. Conversely, the broad halo observed in the range of 10° to 20° indicates the amorphous characteristics of the chitosan (CS) and polycaprolactone (PCL) components, which are known to generate disordered, non-crystalline regions.^45^ The lack of significant shifts in peak positions implies that the drug loading procedure did not disrupt the crystalline architecture of the MOF, suggesting that the encapsulation of DOX occurred through a non-destructive physical process within the pores. Furthermore, the observed decrease in intensity of the MOF peaks relative to pure La-MOF can likely be credited to the dilution effect induced by the polymeric matrix as well as partial obstruction of the crystalline sites. The XRD results collectively substantiate the effective incorporation of nanofiber membrane. This integration results in a composite that is not only structurally stable but also functionally appropriate for applications in pH-responsive drug delivery, as depicted in Fig. 2(a).

#### 3.1.2. N_2_ adsorption/desorption isotherm

The nitrogen adsorption/desorption isotherm for the DOX@La-MOF membrane, illustrated in Fig. 2(b), reveals a type IV isotherm featuring an H3 hysteresis loop. This pattern proposes the attendance of a mesoporous construction considered by slit-shaped pores, which frequently arise from the clustering of plate-like units. The observed gradual and nearly linear increase in the volume of nitrogen adsorbed over the relative pressure range (*P*/*P*_0_ = 0.05–1.0) indicates a consistent mesoporosity throughout the material. The BET-specific surface area of the composite was measured at 68.4 m^2^ g^−1^. This value, while reduced compared to that of the unaltered La-MOF, aligns with predictions regarding surface area loss stemming from the integration of polymeric materials, specifically chitosan and polycaprolactone, which occurred during the electrospinning procedure.^46^ Additionally, the internal encapsulation of DOX within the metal–organic framework (MOF) further contributes to this observed surface area reduction. The identified H3-type hysteresis loop at raised relative pressures serves to substantiate the existence of non-rigid mesopores together with capillary compression effects, which are representative of lamellar pore architectures. Additionally, the measured total pore volume of 0.12 cm^3^ g^−1^ suggests a moderate yet adequate level of porosity, facilitating the diffusion and retention of drug molecules. These findings validate that the structural coherence and porosity of La-MOF were preserved following its incorporation into the nanofibrous membrane and subsequent drug loading, thereby maintaining its operational capacity for applications related to controlled drug delivery.

#### 3.1.3. FT-IR

The FT-IR spectrum of the DOX@La-MOF membrane, presented in Fig. 2(c), offers significant visions into the chemical composition and the effective amalgamation of its various constituents: DOX, La-MOF, CS, and PCL within the composite. A pronounced absorption band 3420–3200 cm^−1^ range can be credited to the combined O–H and N–H stretching vibrations. This finding proposes the existence of hydroxyl and amino groups derived from chitosan, along with hydroxyl groups from DOX and coordinated water molecules within the MOF structure. Moreover, the specific absorption band at 2920 cm^−1^ is related with C–H stretching vibrations from the aliphatic chains present in both PCL and CS. The distinct peak detected at around 1650 cm^−1^ is indicative of the C=O stretching vibration associated with amide (amide I) and carbonyl functional groups. This observation substantiates the presence of DOX and ester linkages stemming from PCL.^47^ Furthermore, the spectral band located near 1540 cm^−1^ is attributed to the N–H bending vibrations, known as amide II, which further corroborates the effective integration of DOX and chitosan into the structure. Additionally, the band found around 1385 cm^−1^ likely resembles to the symmetric stretching of –COO^−^ assemblies, which serves as a distinctive marker for metal–carboxylate coordination within the La-MOF. The prominent absorption bands identified within the fingerprint region, specifically ranging from 1200 to 1000 cm^−1^, are revealing of C–O–C and C–N stretching vibrations emanating from both the polymer backbone and the DOX structure. Additionally, the bands located in the lower wavenumber domain, particularly those between 600 and 500 cm^−1^, are attributed to La–O metal–oxygen bonding vibrations. This observation substantiates the formation of La–MOF and its successful integration into the nanofiber medium. Collectively, the FT-IR spectrum validates the attendance of all functional components within the composite structure, evidencing the successful creation of the DOX@La-MOF membrane. This maintains the structural integrity of each individual component and indicates the potential for regulated drug release through targeted functional interactions.

#### 3.1.4. EDX analysis

The Energy-Dispersive X-ray (EDX) spectrum illustrated in Fig. 2(d) offers an in-depth elemental examination of the DOX@La-MOF nanofiber membrane, validating the effective integration of its primary elements within the electrospun architecture. The spectrum features different peaks attributed to C, N, O, and La, which are critical constituents of the La-MOF structure as well as the polymer matrix made up of chitosan and polycaprolactone. The numerical elemental composition, as depicted by the pie chart included, reveals that oxygen constitutes the largest proportion at 47.7%, succeeded by carbon at 29.2%, nitrogen at 16.3%, and lanthanum at 6.8%. This considerable presence of oxygen is largely due to the carboxyl and hydroxyl functional groups integrated into both the MOF and polymer chains, in addition to the oxygen-dense coordination milieu surrounding La^3+^ ions. Carbon is derived from the organic linkers existing in La-MOF, as well as the structural components of CS and PCL. Meanwhile, the discovery of nitrogen signifies the being of amine practical groups associated with both chitosan and DOX molecules. The identification of lanthanum peaks at various locations provides additional evidence of the effective mixture of the La-MOF within the membrane.^48^ The consistent spreading and comparable strength of these peaks indicate the uniform incorporation of La-MOF within the nanofibrous scaffold. This also suggests that the structural integrity of the functional components essential for drug loading and delivery applications has been maintained.

#### 3.1.5. SEM analysis

The SEM examination of the DOX@La-MOF nanofiber membrane, exemplified in Fig. 2(e), demonstrates a remarkably uniform and interconnected fiber architecture produced through electrospinning. The absence of visible beads or structural imperfections suggests that the processing conditions have been finely tuned. The nanofibers display a smooth and continuous cylindrical form, with diameters measured between roughly 76.0 and 150.0 nm, and an average diameter primarily falling within the range of 90 nm. This results in a narrow size distribution, which is advantageous for maximizing surface area and facilitating effective drug interactions. High-resolution imaging demonstrates that DOX@La-MOF nanofiber membrane particles have been uniformly integrated into a polymeric matrix made of chitosan and polycaprolactone, while preserving the honesty of the nanofiber structure. The lack of particle clustering or surface irregularities indicates effective encapsulation or surface integration of the MOF crystallites within the polymer chains. The resulting porous and intertwined fiber configuration promotes optimal permeability and establishes interconnected channels that may improve drug loading and controlled release capabilities. This specific morphological structure, defined by nanoscale dimensions and consistent dispersion, not only guarantees the mechanical constancy of the membrane but also improves its functional effectiveness as a reliable carrier for targeted and sustained drug delivery systems.^49^

The SEM-EDS elemental mapping showed in Fig. 2(f) offers substantial evidence regarding the effective and uniform integration of key elements, with C, N, O, and La, within the nanofiber membrane structure. The central SEM image substantiates the development of a clearly outlined, bead-free, and intertwined fiber network. Concurrently, the surrounding elemental mappings illustrate a steady and uniform distribution of each of these elements throughout the membrane, reinforcing the consistency of the composition within the nanofibrous material. In a detailed analysis of the composition, carbon constitutes 36%, predominantly sourced from the polycaprolactone and chitosan polymer matrices along with the organic linker present in the La-MOF. The nitrogen percentage, at 10%, can be traced back to the amine groups found in chitosan and the doxorubicin molecules, which suggests an effective drug encapsulation process. Oxygen, making up 32% of the arrangement, arises from the functional groups related with the polymer backbone and the coordination sites existing within the MOF structure. Finally, the presence of lanthanum at 22% provides evidence for the successful combination of La-MOF particles within the fiber matrix. The lack of aggregation or elemental clustering provides additional indication for the uniform dispersion of La-MOF and the drug within the electrospun membrane. This consistent elemental distribution is essential for optimizing the membrane's functional efficacy in drug delivery, as it promotes uniform drug release, maintains structural integrity, and facilitates effective responses to external stimuli. Consequently, this finding substantiates the successful design and fabrication of the DOX-loaded La-MOF nanofiber system.^49^

#### 3.1.6. XPS analysis

**Fig. 3:**
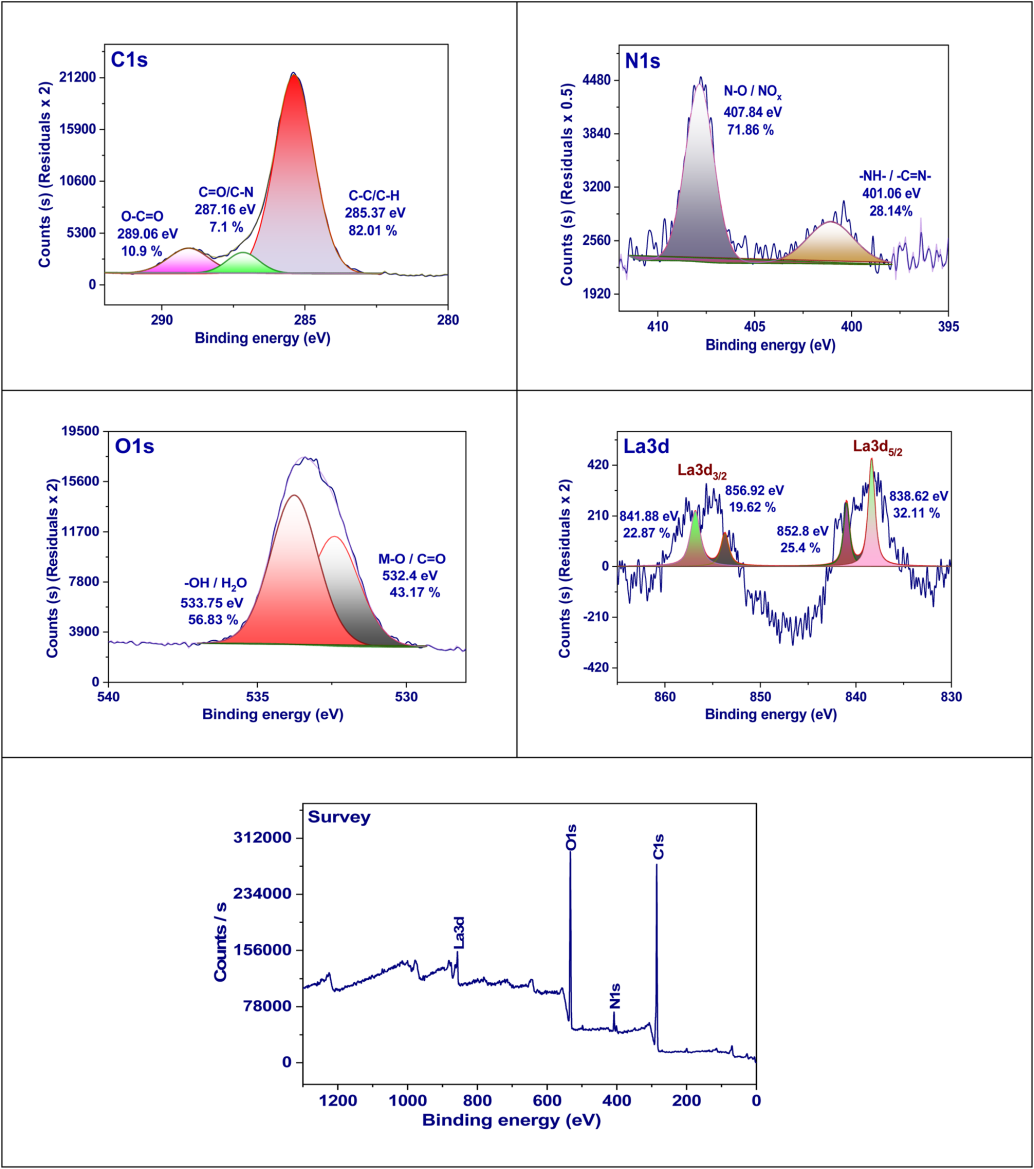
XPS pattern of DOX@La-MOF.

The high-resolution X-ray Photoelectron Spectroscopy (XPS) spectrum of the C 1s area for the DOX@La-MOF nanofiber membrane proposals a inclusive analysis of the chemical composition and bonding environments of carbon atoms confidential the composite structure (Fig. 3). The spectrum was deconvoluted into three separate peaks, indicating the presence of various useful groups. The most prominent peak, appearing at a binding energy of 285.37 eV and representing 82.01% of the total area, is credited to C–C and C–H bonds typically found in aliphatic carbon chains and aromatic rings, highlighting the organic backbone constituted by PCL, CS, and aromatic linkers within the MOF network. The second peak, situated at 287.16 eV and financing for 7.1% of the total carbon content, correlates with carbon in C=O besides C–N bonding environments, suggesting the presence of amide and imine groups. The third peak, at 289.06 eV and representative 10.9% of the carbon signal, is associated with O–C=O bonds, indicative of ester groups. Overall, the XPS C 1s analysis validates the effective incorporation and chemical integration of MOF components with CS and PCL, supporting the multifunctional potential of the nanofiber membrane for drug loading and sustained release applications.^50^

The XPS study of the N 1s region for the DOX@La-MOF membrane provides essential visions into the nitrogen functionalities that are incorporated into its composite structure (Fig. 3). The deconvoluted spectrum displays two main peaks, which correspond to different nitrogen chemical states. The prominent peak at 407.84 eV accounts for 71.86% of the total nitrogen content and is related with protonated or else oxidized nitrogen species. These species may result from the coordination of nitrogen atoms with La^3+^ metal centers or from partial oxidation that occurs during the synthesis or drug loading processes. The presence of these nitrogen functionalities indicates a strong interaction with the MOF framework, potentially enhancing the structural stability and drug-binding capacity of the membrane.^51,52^ The second peak, noted at 401.06 eV and contributing 28.14% to the total nitrogen content. These groups are likely formed from chitosan during the membrane's functionalization process. These nitrogen functionalities play a essential role in the interaction with DOX *via* hydrogen bonding or coordination, thereby improving drug encapsulation and facilitating controlled release. Overall, the XPS N 1s spectrum verifies the effective incorporation of nitrogen-containing groups within the DOX@La-MOF, highlighting their important contributions to both structural integration and the membrane's performance in drug delivery.^53^

The XPS spectrum of the O 1s region for the DOX@La-MOF membrane reveals important details regarding the oxygen-containing functional groups and their respective chemical states (Fig. 3). The deconvoluted spectrum features two primary peaks, each representing different oxygen types present in the composite. The first peak, detected at 533.75 eV and constituting 56.83% of the overall oxygen content, is linked to oxygen atoms within hydroxyl (–OH) groups and/or adsorbed water molecules. These are typically found in conjunction with chitosan and polycaprolactone matrices, as well as within the hydrated coordination environment surrounding La^3+^ ions in the MOF. This finding indicates that a substantial fraction of the oxygen exists in labile or hydrogen-bonded forms, suggesting a potential role in drug interactions *via* hydrogen bonding. The second peak, recorded at 532.4 eV and financing for 43.17% of the oxygen content, is related with M–O or C=O groups. This proposes the attendance of coordinated oxygen species integral to the La–O bond network within the MOF structure, or ester groups originating from PCL. These oxygen species are essential for structural integrity and the coordination of DOX molecules. The identification of both chemisorbed and physisorbed oxygen further confirms the actual integration of organic polymers with the inorganic La-MOF framework and underscores the vital role of oxygen functionalities in influencing the hydrophilicity, stability, and drug-binding capacity of the nanofiber membrane.^54^

The XPS spectrum of La 3d obtained from the DOX@La-MOF membrane offers important visions into the oxidation states and coordination situation of lanthanum inside the framework (Fig. 3). The analysis shows several deconvoluted peaks corresponding to the La 3d_5/2_ and La 3d_3/2_ spin–orbit components, with each component exhibiting characteristic doublets. Specifically, the peaks at 838.62 eV (32.11%) and 852.8 eV (25.4%) are associated with La 3d_5/2_, while those at 841.88 eV (22.87%) and 856.92 eV (19.62%) relate to La 3d_3/2_. These binding energy values align with the La^3+^ oxidation state, indicating that lanthanum is present in a trivalent form within the MOF. The observation of multiple chemical environments, as suggested by the peak splitting, implies a heterogeneous coordination environment, likely due to interactions between La^3+^ ions and organic ligands that contain oxygen or nitrogen donor atoms (*e.g.*, hydroxyl, or amine groups).^54^ This intricate coordination structure contributes to the chemical stability and practical efficacy of the DOX@La-MOF membrane, reinforcing its applicability in drug encapsulation and controlled release mechanisms. Consequently, the La 3d spectrum confirms the effective combination of the La-based MOF network and its incorporation into the nanofibrous membrane (Fig. 3).

### 3.2. Controlled release of drugs

#### 3.2.1. Effect of pH

**Fig. 4:**
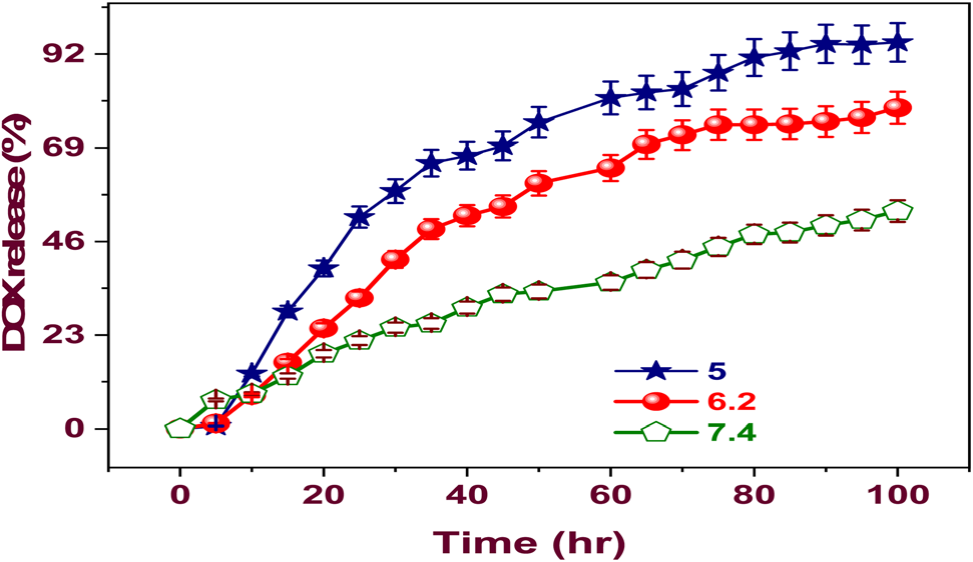
Proportion of drug release at various pH levels.

The DOX@La-MOF membrane exhibited a drug loading capacity (DLC) of 86.4% alongside an encapsulation efficiency (EE) of 94.2%. These figures suggest that the La-MOF displays a high efficiency in the encapsulation of DOX.^55^ To evaluate the release properties more comprehensively, a batch of experiments was conducted at three distinct pH values (7.4, 6.2, and 5.0), while keeping the temperature consistently at 25 °C for a duration of 140 hours. Fig. 4 shows the outcomes of these investigations. The results clearly demonstrate a DOX release pathway from the membrane that is sensitive to pH variations. The release rate is noticeably higher at pH 5.0 than at pH values of 7.4 and 6.2, importance the membrane's sensitivity to pH changes. Over the course of a 140 hour evaluation period, the quantifiable release rates of DOX from the La-MOF nanofiber membrane were recorded at 53.48, 78.8, and 94.9% across pH levels of 7.4, 6.2, and 5.0, individually. The release kinetics observed indicate that the La-MOF nanofiber membrane possesses a pronounced ability to improve the release of drugs when in proximity to tumor locations, facilitating internalization by cancer cells. The acidic microenvironment that is often present in cancerous tissues is what causes this impact. As a result, this process's pH-sensitive features increase DOX's cytotoxicity, representative a possible advancement in advantageous plans (Fig. 4).

The drug release kinetics are significantly influenced by the hydrogen bonding that occurs among DOX and La-MOF membrane. Particularly, DOX release is meaningfully higher at pH 5.4 than it is at pH 7.4. This finding suggests that DOX is released more efficiently in tumor-like settings, which is especially pertinent considering the acidic nature of the tumor micro-environment. A process like this is beneficial since it may lessen the harm to nearby healthy tissues. The variations in the intensity of hydrogen bonding contacts among the practical groups of DOX (–NH_2_ and –OH) and the La-MOF membrane can account for the release's pH sensitivity.^55^ The increased hydrogen bonding connections at neutral pH lead to a lower rate of DOX release under these circumstances.^56^ Hydrogen interactions through bonds have been found to be stronger in basic settings than in acidic ones. In acidic conditions, this difference in strength causes a greater release of DOX. Through the process of endocytosis, the drug-loaded nanocarrier enters the tumor cell after being directed there by an external magnetic field. Hydrogen bonds among DOX and the La-MOF membrane are broken when the amine groups in DOX are protonated. The medicine releases more quickly as a result of this disturbance. Furthermore, Fig. 4 demonstrates that a significantly greater quantity of DOX is released at body temperature as opposed to room temperature.

#### 3.2.2. Effect of temperature

**Fig. 5:**
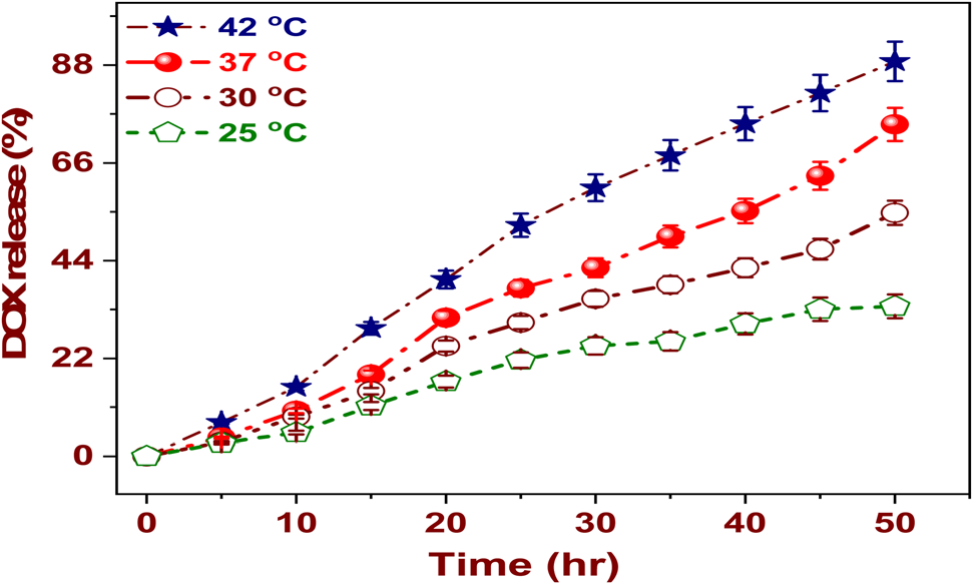
Temperature's impact on the proportion of medication release.

The influence of temperature on the release kinetics of DOX@La-MOF nanofiber membrane was meticulously analyzed across four different temperatures: 25, 30, 37, and 42 °C, over a 50 hour duration, as illustrated in Fig. 5. The findings indicate a pronounced temperature dependency in the release rate of DOX, with elevated temperatures markedly enhancing the release dynamics. At a temperature of 25 °C, the cumulative release was confined to roughly 45%, which suggests a relatively sluggish diffusion of DOX since the nanofiber medium. However, as the temperature was raised to 30 °C and subsequently to 37 °C, there was a significant increase in release efficiency, with values reaching about 60 and 75%, respectively.^56^ Particularly striking is the observation at 42 °C, a temperature that simulates hyperthermic conditions typical in tumor environments, where the cumulative release surpassed 90%. This underscores the thermally responsive characteristic of the nanofibers. The observed temperature-induced increase in drug release can be explained by multiple significant factors. Firstly, elevated temperatures enhance the molecular mobility, which, in turn, accelerates the diffusion of DOX through the porous framework of the DOX@La-MOF nanofiber membrane. Secondly, the thermal expansion of the nanofiber matrix is likely to augment both pore size and permeability. Lastly, the thermal effects lead to a diminished strength of hydrogen bonding besides coordination connections between DOX and the useful groups inherent to the MOF structure. This reduction in binding affinity ultimately facilitates the process of drug desorption. The temperature-responsive release mechanism presents significant benefits in the context of cancer treatment. By utilizing localized hyperthermia, it enables the precise initiation of drug release at targeted sites, thereby reducing systemic side belongings and enhancing the overall efficacy of the treatment. This observation highlights the potential of the DOX@La-MOF as an advanced drug delivery organization that is both thermally responsive and capable of bringing chemotherapy in a measured and targeted manner.

### 3.3. Kinetic models

The application of kinetic models provides crucial insight into the kinetics of DOX@La-MOF nanofiber membrane release. This approach facilitates the elucidation of the fundamental mechanisms governing the release process while simultaneously supporting the refinement of the drug transfer system.^57^ The zero-order model, characterized by its premise of a constant release rate that remains unaffected by drug concentration, is particularly effective for sustaining steady-state therapeutic concentrations. This model is indicative of a controlled release manner when a linear relationship manifests in the data. In dissimilarity, the first-order model proposes that the release rate is influenced by concentration, suggesting that as the quantity of DOX within the membrane decreases over time, so too does the rate of its release. The model of Higuchi is utilized to designate a release mechanism that is dependent on the time squared, founded on Fickian diffusion, and is frequently used in matrix-based systems.^58^ A strong correlation with this model signifies that the diffusion of DOX over the (CS/PCL) matrix and the La-MOF nanofiber membrane is the primary release mechanism (Table S7). The Korsmeyer–Peppas model provides a more comprehensive analysis, particularly when the release process's specifics are ambiguous.^59^ This model employs the release exponent ‘*n*’, which may involve processes related to polymer swelling or relaxation. Finally, the Hixson–Crowell model addresses the evolution of surface area and particle size with time, reflecting phenomena such as erosion or degradation of the matrix, and a strong fit to this model indicates that structural changes within the nanofiber membrane play a role in the release characteristics. In summary, these kinetic models not only clarify the release performance of DOX but also support the effective design of nanofiber-based drug delivery systems aimed at improving therapeutic outcomes.

**Fig. 6:**
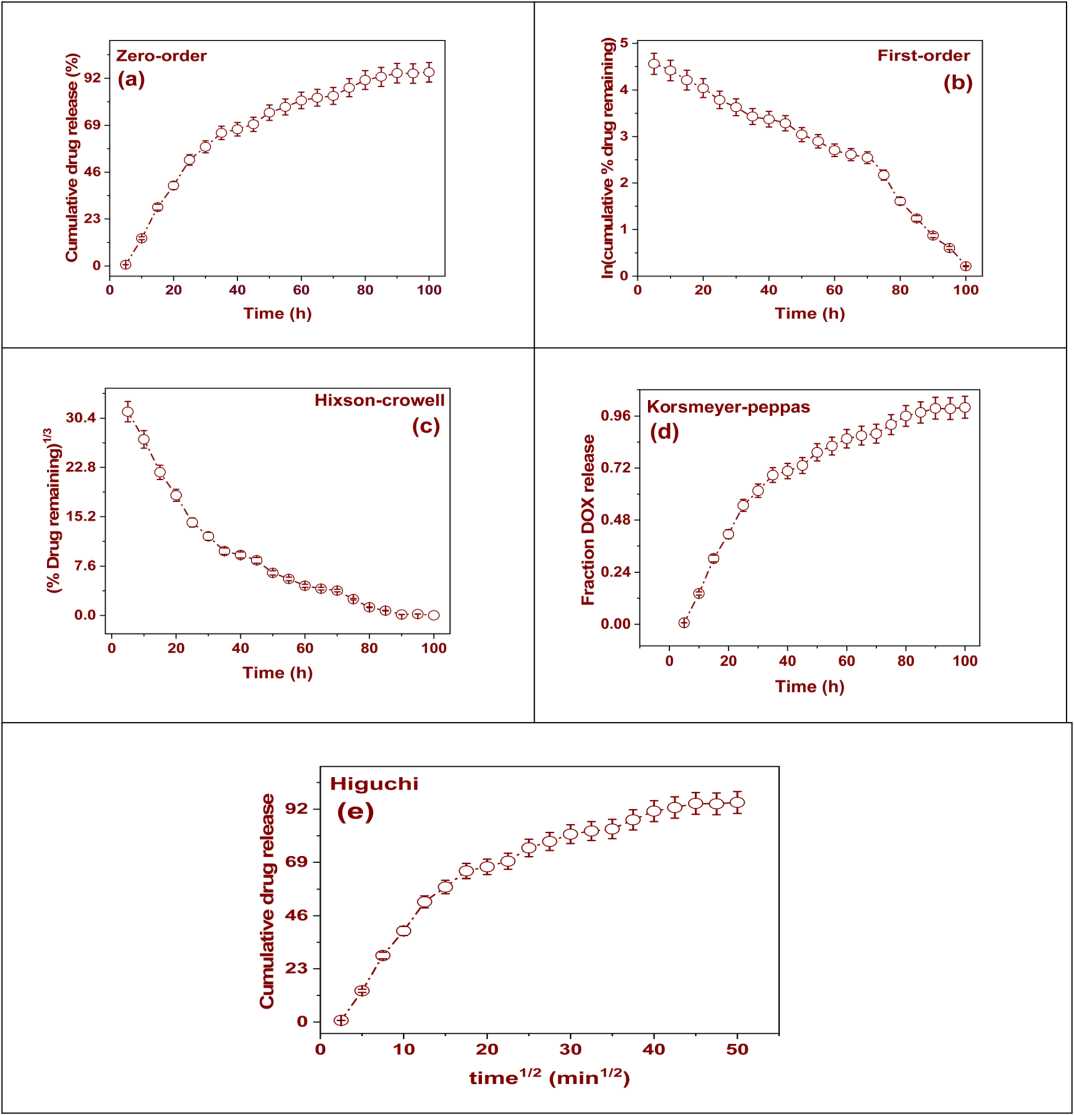
Kinetic models for DOX release: (a) zero-order model, (b) first-order model, (c) Hixson–Crowell model, (d) Korsmeyer–Peppas model, and (e) Higuchi model.

The progressive release profile of DOX generated by the DOX@La-MOF nanofiber membrane, as determined by the zero-order kinetic equation, is shown in Fig. 6(a). This equation indicates a consistent rate of drug release that remains unaffected by alterations in drug concentration, a characteristic that is beneficial for sustaining stable therapeutic concentrations over extended durations. The release curve exhibits a steady increment in DOX liberation, culminating in roughly 92% release over a period of 100 hours. The almost linearity of the data aligning with the zero-order model further substantiates the behavior of controlled and sustained release.^57^ The determined zero-order release degree constant (*K*_0_) was found to be 1.39 h^−1^, signifying that a consistent 1.39% of the encapsulated DOX is released each hour. This particular release characteristic reflects the ability of the membrane to efficiently control drug diffusion, thereby yielding a prolonged release profile. Moreover, the existence of error bars underscores the reproducibility and reliability of the experimental results, reinforcing the potential of the membrane as an effective stage for sustained distribution of anticancer agents.

Fig. 6(b) illustrates the first-order kinetic modeling of DOX release from the membrane. According to this modeling, the amount of medication that is still present in the structure directly correlates with the rate of release. Plotting the percentage of drug natural logarithm against time using this analytical framework yields a linear association that validates the first-order kinetics theory. The data points demonstrate a steady decline in the logarithmic concentration of DOX over a period of 100 hours, indicating a decreasing rate of release as the drug concentration in the membrane diminishes. The slope of the fitted line represents the first-order release degree constant (*K*_F_), which has been designed to be 0.40 h^−1^. The data suggests that the release mechanism adheres to an exponential decay model, which is characteristic of systems where drug diffusion is influenced by the concentration gradient. The observed linear decline indicates a progressive DOX molecules release from the nanofiber matrix, with a noticeable slowing in the release rate as the reservoir diminishes. The inclusion of error bars indicates a great degree of repeatability in the repeated measurements. In summary, this kinetic profile elucidates the diffusion characteristics of DOX from the hybrid nanofibrous construct, reinforcing its potential applicability for controlled drug delivery systems that respond to concentration changes (Table S7).

Fig. 6(c) illustrates the kinetics of DOX release from the DOX@La-MOF membrane, analyzed through the Hixson–Crowell model, which characterizes drug release mechanisms associated with alterations in surface area as well as particle morphology throughout the dissolution procedure. The cube root of the residual drug% is plotted *versus* time in this model, and the resulting curve shows a slow fall that suggests continuous erosion through the nanofibrous network. The observed linear correlation, especially during the initial to mid-releases phases, supports the conclusion that the release dynamics are primarily influenced by surface area-dependent mechanisms. This phenomenon indicates that as the drug diffuses out, the matrix experiences a continuous reduction in size. The Hixson–Crowell release rate constant (*K*_HC_) was determined to be 0.289 h^−1^, which reflects the diminishing effective surface area of DOX within the membrane over time. This finding implies that both diffusion processes and structural degradation play roles in the drug release mechanism. The presence of error bars on the data points serves to validate the reproducibility of the measurements, thereby enhancing the credibility of the release profile. Overall, these results underscore the crucial roles of matrix erosion and dimensional reduction in controlling DOX release from the membrane, thereby highlighting its probable application in sustained and geometry-responsive drug transfer systems.

Fig. 6(d) gifts the kinetics of DOX release from the membrane, investigated over the model of Korsmeyer–Peppas a widely utilized framework for assessing release mechanisms in polymeric and porous resources. In this model, the released fraction of DOX is graphed against time, revealing a sustained and gradual increase over a duration of 100 hours, approaching nearly complete release. The calculated value of *n* was determined to be 0.00915, which is significantly lower than the conventional threshold indicative of Fickian or anomalous diffusion. This finding implies that the release mechanism aligns more closely with a nearly zero-order kinetic pattern, rather than being predominantly diffusion-controlled. The release rate constant (*K*_F_) was resolute to be 0.23 h^−1^, representative a moderate and stable release rate throughout the experiment. The gradual increase of the release curve, along with the inclusion of error bars, corroborates the reproducibility and dependability of the release data collected. In conclusion, the Korsmeyer–Peppas model designates that the membrane facilitates a sustained and regulated drug release process, chiefly inclined by the matrix properties and demonstrating quasi-zero-order kinetics due to restricted diffusion within the membrane framework.

Fig. 6(e) offerings an analysis of the release kinetics of DOX from the membrane, evaluated through the lens of the model of Higuchi. This model suggests that the drug release procedure is ruled by diffusion, where the rate is directly related to the square root of elapsed time. The graph depicts the cumulative percentage of DOX released planned against the time squared (min^1/2^), revealing a characteristic initial phase of rapid drug release, which transitions into a more gradual and sustained diffusion phase, a behavior commonly observed in porous matrix systems.^58^ The detected linear relationship during the initial to mid-development phases substantiates the validity of the employed model. The derived Higuchi constant (*K*_H_) is 1.74, suggesting a notably elevated rate of diffusion. This figure underscores the porous and interlinked architecture of the nanofiber matrix, which aids in promoting effective drug transport. Additionally, the incorporation of error bars further supports the reproducibility and reliability of the experimental results. In summary, the findings confirm that the release of DOX from the membrane is predominantly regulated by a diffusion-controlled mechanism, making it particularly appropriate for scenarios necessitating consistent and predictable drug delivery.

The analysis of the kinetic modeling data regarding DOX release from the membrane, as presented in Table S8, reveals a clear concentration-dependent mechanism characterized by the first-order kinetic model. This model is distinguished by its lowest reduced Chi-square value of 1.40883, a minimal residual sum of squares measuring 0.97737, and a high correlation coefficient of *R*^2^ = 0.95526. These metrics indicate that this model provides a better fit compared to alternative models. The implications of these results suggest that the DOX release rate is influenced chiefly by its remaining concentration within the nanofiber matrix, which is consistent with a diffusion-controlled mechanism. According to this process, the release rate would eventually slow down as the DOX quantity drops. The zero-order model demonstrated a notable *R*^2^ value of 0.9087; however, its elevated reduced Chi-square (675.61915) and substantial residual error diminished its effectiveness in accurately depicting the actual release dynamics. The model of Korsmeyer–Peppas, which is commonly employed to elucidate the release mechanism, produced an “*n*” value of 0.00915 and an *R*^2^ of 0.85763, signifying a Fickian diffusion process. Despite this indication, the model did not achieve the statistical fitness characteristic of the first-order model. Moreover, both the Hixson–Crowell and Higuchi models exhibited comparable *R*^2^ values of 0.85763, yet they were associated with considerably higher reduced Chi-square values (231.45299 and 2083.07687, respectively), rendering them less suitable for this particular system. Collectively, these findings substantiate that the DOX release from the membrane primarily adheres to first-order kinetics, thereby implying a controlled diffusion process driven by concentration, which is optimal for sustained and responsive drug delivery applications.^59^

### 3.4. Release stages

**Fig. 7:**
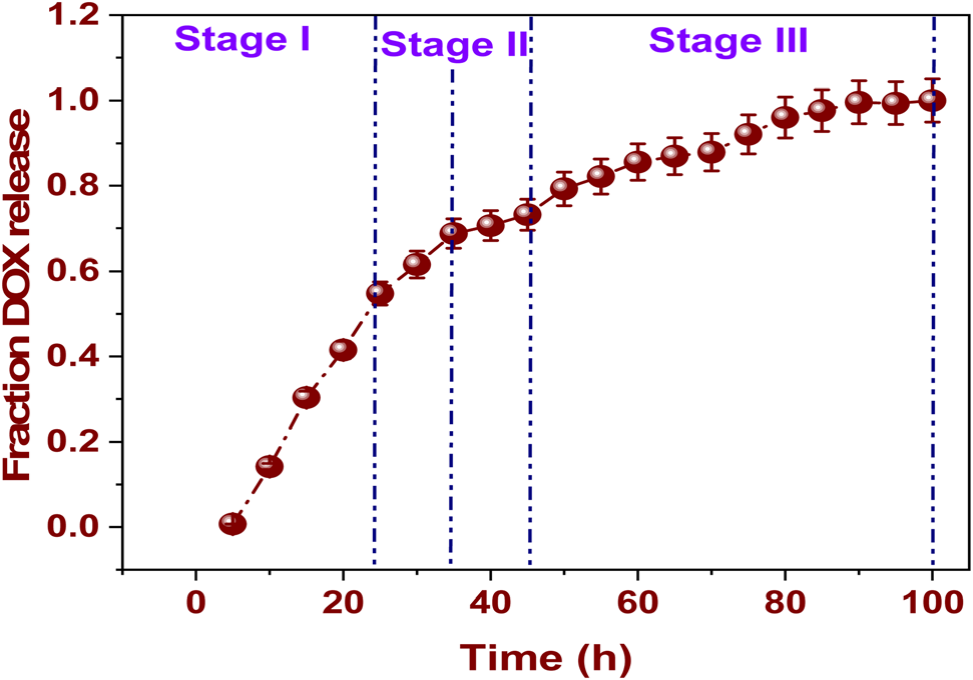
The membrane releases DOX fractionally over time.

Fig. 7 depicts the cumulative, time-dependent profile of DOX release from the membrane, highlighting a clearly defined three-phase kinetic behavior: stage I, stage II, and stage III. On the *Y*-axis, the fraction of released DOX is indicated, ranging from 0.0 to 1.0 (representing 0.0 to 100% release), while the *X*-axis presents the release duration, extending to nearly 100 hours. This graph offers essential insights into the dynamics governing the DOX release from the membrane.^60^

#### 3.4.1. Stage I: the first step of burst release

The period spanning from 0 to 25 hours signifies the initial phase of burst release, which is marked by a rapid and significant escalation in the DOX release profile. This expedited release can be primarily ascribed to the desorption of DOX particles that are either weakly attached or located on the exterior of the membrane (Fig. 7). The notable rate of release observed during this phase is influenced by a pronounced concentration gradient between the drug-entrapped matrix and the adjacent release medium, thereby promoting the swift diffusion of DOX into the surrounding solution.^61^

#### 3.4.2. Stage II: the sustained step release

The period spanning 25 to 50 hours signifies the sustained release phase, characterized by a marked reduction in the rate of DOX release. Throughout this stage, the release mechanism is mainly governed by the steady diffusion of DOX molecules from the internal porous architecture of the La-MOF framework (Fig. 7). This pattern suggests that the drug is securely retained within the complex network of the MOF, progressively moving through the interlinked nanopores and fibrous structure of the nanofiber membrane. As a consequence, this leads to a regulated and extended drug release profile.^62^

#### 3.4.3. Stage III: the equilibrium or plateau phase

The time frame extending from 50 to 100 hours represents the concluding equilibrium or plateau phase in the release profile. During this period, there is a gradual stabilization in the release rate, signifying that a significant portion of the encapsulated DOX has been released (Fig. 7). The relatively constant fraction of release observed at this stage implies that the residual DOX is either tightly bound within the framework of the MOF structure or that the external medium has attained saturation levels. This phenomenon emphasizes the strong structural honesty of the membrane and validates its effectiveness in facilitating a stable and regulated drug release over an extended duration.^63^

The three-phase release profile presented demonstrates the sophisticated and adjustable release characteristics of the DOX@La-MOF nanofiber system. The system initiates with a swift release phase, facilitating immediate therapeutic intervention. This is followed by a sustained release phase, which ensures an extended drug presence, culminating in an equilibrium phase characterized by negligible premature drug leakage. Such a regulated and sequential drug release mechanism is particularly beneficial in oncology treatments, where maintaining consistent dosage, minimizing adverse effects, and achieving targeted release are essential for optimizing therapeutic effectiveness and safeguarding patient well-being (Table 2).

**Table 2:** Timelines for the various release phases and DOX's numerical values (three phases)

Stage	Time interval (h)	Mechanism	Description
I	0–25	Initial burst release phase	Quick surface bond release DOX
II	25–50	Sustained release phase	Matrix diffusion-based slow internal release
III	50–100	Equilibrium or plateau phase	Prolonged release as a result of membrane damage

### 3.5. Statistical analysis

#### 3.5.1. ANOVA

The ANOVA results for the quadratic model that describes the release action of DOX from DOX@La-MOF membranes are shown in Table 3. A strong connection among the investigational factors and the response variable is suggested by the statistical evaluation, which shows that the model has importance with a high F-value of 135.09 and a *p*-value of below 0.0001.^24^ The model's determination coefficient (*R*^2^ = 0.9943), adjusted *R*^2^ (0.9869), and predicted *R*^2^ (0.9084) designate an exceptional goodness-of-fit and strong predictive capability, confirming its applicability in describing DOX release behavior. An adequate precision score of 34.2209 indicates a high signal-to-noise ratio, significantly surpassing the threshold of 4, thus reinforcing the model's reliability.^64^ The examination of individual influences reveals that time (*B*) has the most significant impact (*F* = 826.07), followed by temperature (*A*) and pH (*C*), with all factors yielding *p*-values lower 0.0001, underscoring their essential roles in DOX release. Furthermore, the contact terms *AB*, *AC*, and *BC* also demonstrated significance through *p*-values of 0.0038, 0.0226, and 0.0059, individually, highlighting that the combined belongings of these variables significantly influence the release mechanism. The quadratic term *B*^2^ was originate to be noteworthy (*p* < 0.0001), indicating a nonlinear effect of time on the release profile, while terms *A*^2^ and *C*^2^ were deemed insignificant, suggesting a nearly linear relationship for temperature and pH within the tested range.^65,66^ Additionally, the low residual error (64.26), the absence of pure error, and a minimal standard deviation (3.03) further validate the reliability and dependability of the investigational data. In conclusion, the ANOVA outcomes strongly endorse the efficacy of the proposed model in optimizing and predicting DOX release from the membrane.^64^

**Table 3:** Variance examination to evaluate the models' suitability

Source	Squares amount	df	Square mean	*F*-value	*p*-value	
Model	11 161.34	9	1240.15	135.09	<0.0001	Significant
*A*-temperature	561.29	1	561.29	61.14	0.0001	
*B*-time	7583.30	1	7583.30	826.07	<0.0001	
*C*-pH	525.76	1	525.76	57.27	0.0001	
*AB*	166.26	1	166.26	18.11	0.0038	
*AC*	77.81	1	77.81	8.48	0.0226	
*BC*	139.99	1	139.99	15.25	0.0059	
*A* ^2^	0.0939	1	0.0939	0.0102	0.9223	
*B* ^2^	2085.41	1	2085.41	227.17	<0.0001	
*C* ^2^	2.57	1	2.57	0.2805	0.6128	
Residual	64.26	7	9.18			
Lack of fit	64.26	3	21.42			
Pure error	0.0000	4	0.0000			
Cor total	11 225.60	16				
*R* ^2^	0.9943					
*R*_Adj_^2^	0.9869					
*R*_Pred_^2^	0.9084					
Adeq. precision	34.2209					
Std. dev.	3.03					
Mean	43.02					
C.V.%	7.04					

A comparison of the sum of squares for the linear, quadratic, cubic, as well as two-factor interaction (2FI) regression models is shown in Table 4. The purpose of this investigation is to determine the best model for describing how DOX releases from the membranes.^67^ Among the models analyzed, the quadratic model showed the best statistical performance, highlighted by a highly significant sequential *p*-value of less than 0.0001, an *R*_Adj_^2^ value of 0.9869, and *R*_Pred_^2^ of 0.9084. According to these measures, the quadratic model is the greatest choice for handling the intricacies of the drug release process since it provides a good match and exceptional predictive power. In comparison, the linear model, although it yielded a important *p*-value of 0.0002, presented a lower *R*_Adj_^2^ of 0.7198 and *R*_Pred_^2^ of 0.5733, indicating its inadequacy in accounting for the variability of the response.^68–70^ Furthermore, the performance of the 2FI model was even less satisfactory, as it exhibited a non-significant *p*-value of 0.6356, an *R*_Adj_^2^ of 0.6905, and a very low *R*_Pred_^2^ of 0.2134, which suggests it fails to consider crucial interaction and curvature elements in the system. The cubic model was deemed unusable due to being aliased from having zero degrees of freedom, rendering it statistically unreliable. Consequently, the results affirm the quadratic model as the most statistically reliable and predictive method for enhancing the release of DOX from the membrane.^67^

**Table 4:** Sum of squares for the subsequent models

Source	Sum of squares	df	Mean square	Sequential *p*-value	Adjusted *R*^2^	Predicted *R*^2^	
Linear	2555.24	9	283.92	0.0002	0.7198	0.5733	
2FI	2171.19	6	361.86	0.6356	0.6905	0.2134	
Quadratic	64.26	3	21.42	<0.0001	0.9869	0.9084	Suggested
Cubic	0.0000	0			1.0000		Aliased

#### 3.5.2. Cubic communication and perturbation plot

**Fig. 8:**
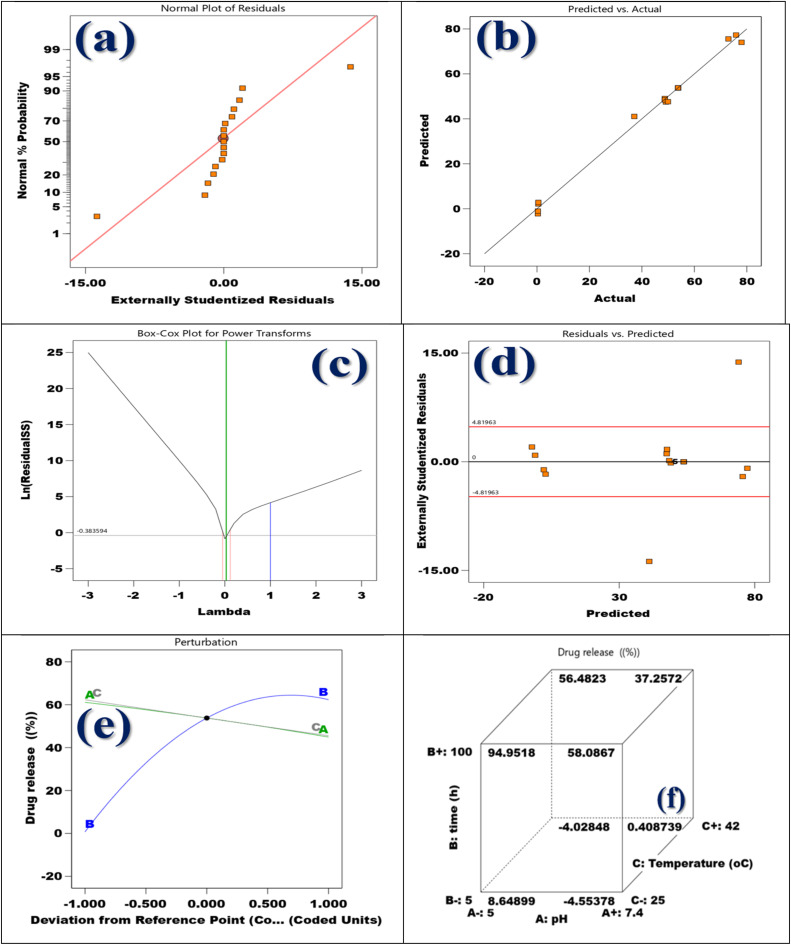
(a) The graph demonstrating the correlation among normal % probability, (b) predicted *vs.* actual (c) power transforms Box–Cox plot for DOX release, (d) highly normalized residuals in contrast to the predicted, (e) perturbation plot, and (f) cubic conversation.

The normal probability plan of the residuals for the DOX release from the membrane indicates that the externally studentized residuals predominantly align with a straight line. This arrangement suggests that the residuals can be considered approximately normally distributed. Such a conclusion implies that the kinetic model employed is appropriate fit for the experimental data and that the assumption of normality an important consideration for the validity of statistical regression models is largely met. The majority of the orange square information points are located near the red comparison line, which further supports the model's suitability.^71^ Nevertheless, there are two data points, one at each extreme of the distribution, that exhibit significant deviation from the line. This discrepancy may indicate the presence of potential outliers or variations in the experimental process that could have affected these particular results. In spite of these exceptions, the overall trend substantiates that the model effectively represents the statistical appearances of DOX release behavior from the membrane system as exemplified in Fig. 8(a).

The DOX release from the DOX@La-MOF nanofiber membrane's predicted *vs.* actual plot, shown in Fig. 8(b), shows a significant correlation among the model's predictions and the experimental (actual) data. The diagonal line, which represents the ideal scenario in which projected values precisely match the experimental data, is closely aligned with the orange square spots. This close alignment proposes that the selected kinetic model successfully replicates the drug release dynamics from the La-MOF system. While minor discrepancies in a few points are noted, these are anticipated due to natural experimental variations and do not materially affect the general effectiveness of the model. The tight clustering and near-linear spread of the data further endorse the model's predictive accuracy, affirming its dependability in characterizing the kinetics of DOX release.^72–74^ Consequently, this plot serves as strong evidence that the utilized mathematical model effectively encapsulates the release mechanism and can be reliably employed for predictive applications in comparable drug delivery systems.^71^

The release of DOX from the membrane is analyzed using the Box–Cox plot shown in Fig. 8(c), which focuses on the optimal transformation needed to stabilize variance and improve the normality of the model's residuals. The plot displays the residual sum of squares' natural logarithm [ln(residual SS)] on the *Y*-axis, while the lambda (*λ*) values used in the Box–Cox transformation procedure are exhibited on the *X*-axis. The vertical green line, which represents the ideal lambda value close to zero, is an important aspect of the plot. This suggests that implementing a log-transformation on the response mutable could improve the data's linearity and homoscedasticity. Additionally, the vertical red and blue lines indicate the 95% confidence interval for *λ*. Notably, the value of 1 (which would imply that no transformation is necessary) lies outside this confidence interval, indicating the need for a transformation in this dataset. The optimal lambda value is identified as approximately −0.38, further confirming that the original data does not conform to a normal distribution and would benefit from a power transformation. Therefore, the analysis emphasizes the importance of applying a Box–Cox transformation to the release data to enhance the model fit and satisfy regression requirements.^75^

The residuals *versus* predicted plot for the DOX release from the membrane, illustrated in Fig. 8(d), acts as a analytical tool to evaluate the quality and underlying suppositions of the kinetic model working. The externally analyzed residuals, which have been adjusted for the impact of each inquiry, are exposed on the *Y*-axis, while the *X*-axis shows the anticipated values for DOX release. In order to demonstrate uniformity (constant variance) and a strong model fit, these residuals should ideally be randomly distributed about the zero line with no obvious pattern.^76–78^ In this analysis, most residuals are contained within the ±4.82 range (denoted by red lines), suggesting an acceptable level of variability. Nonetheless, a few residuals appear significantly outside this range, particularly one extreme point below −15 and another above +15, signaling potential outliers or influential data points. These anomalies may result from experimental errors or changes in the release dynamics that the current model fails to capture entirely. Despite these scattered deviations, the main concentration of residuals near zero implies that the majority of the data aligns well with the model, thereby affirming its general applicability in characterizing the drug release behavior from the La-MOF nanofiber membrane system.^75^

The perturbation plot depicted in Fig. 8(e) reveals the individual impacts of various formulation or process variables on the release of DOX percentage from the membrane. The *X*-axis delineates the coded values for each variable (*A*, *B*, and *C*), which are measured against a central reference point indicated by the black dot, while the *Y*-axis displays the corresponding drug release percentages. Variable *B* demonstrates a distinct curvature, suggesting a significant and nonlinear effect on drug release; initial increases in *B* lead to a marked enhancement in DOX release, but this effect stabilizes or slightly diminishes at elevated levels. Conversely, variables *A* and *C* present nearly horizontal, linear trajectories, indicating limited or negligible effects on drug release within the examined range. The negative slopes for *A* and *C* indicate a slight antagonistic influence, albeit not substantial. This analysis underscores that among the variables evaluated, factor *B* emerges as the most crucial parameter affecting the drug release profile, warranting prioritization in formulation optimization to improve DOX release efficacy from the La-MOF nanofiber membrane system.^71^

The joint impact of three crucial variables pH (*A*), release time (*B*), and temperature (*C*) on the amount of DOX released from the membrane are shown graphically in the 3D cube plot exposed in Fig. 8(f). With the corresponding DOX release percentages displayed, each vertex of the cube represents a distinct mix of low (–) and high (+) amounts of these factors. The analysis reveals that release time (*B*) has the most profound impact, with the highest drug release observed at 94.95% after the maximum duration of 100 hours, combined with low pH (5.0) and a moderate temperature (25 °C). In contrast, the lowest drug release percentage of −4.03% is recorded at the minimum time of 5 hours, at a high pH (7.4), and low temperature (25 °C), which suggests unfavorable release conditions. Additionally, the plot indicates that a lower pH enhances drug release, likely due to mechanisms such as protonation that promote MOF degradation or enhance the solubility of DOX, while elevated temperatures (42 °C) appear to slightly hinder release under certain scenarios. In summary, this cube plot illustrates that optimal DOX release occurs with longer exposure times, acidic pH conditions, and moderate temperatures, stress the consequence of these limits for optimizing therapeutic efficacy in controlled release systems.

#### 3.5.3. Model adequacy verification

**Fig. 9:**
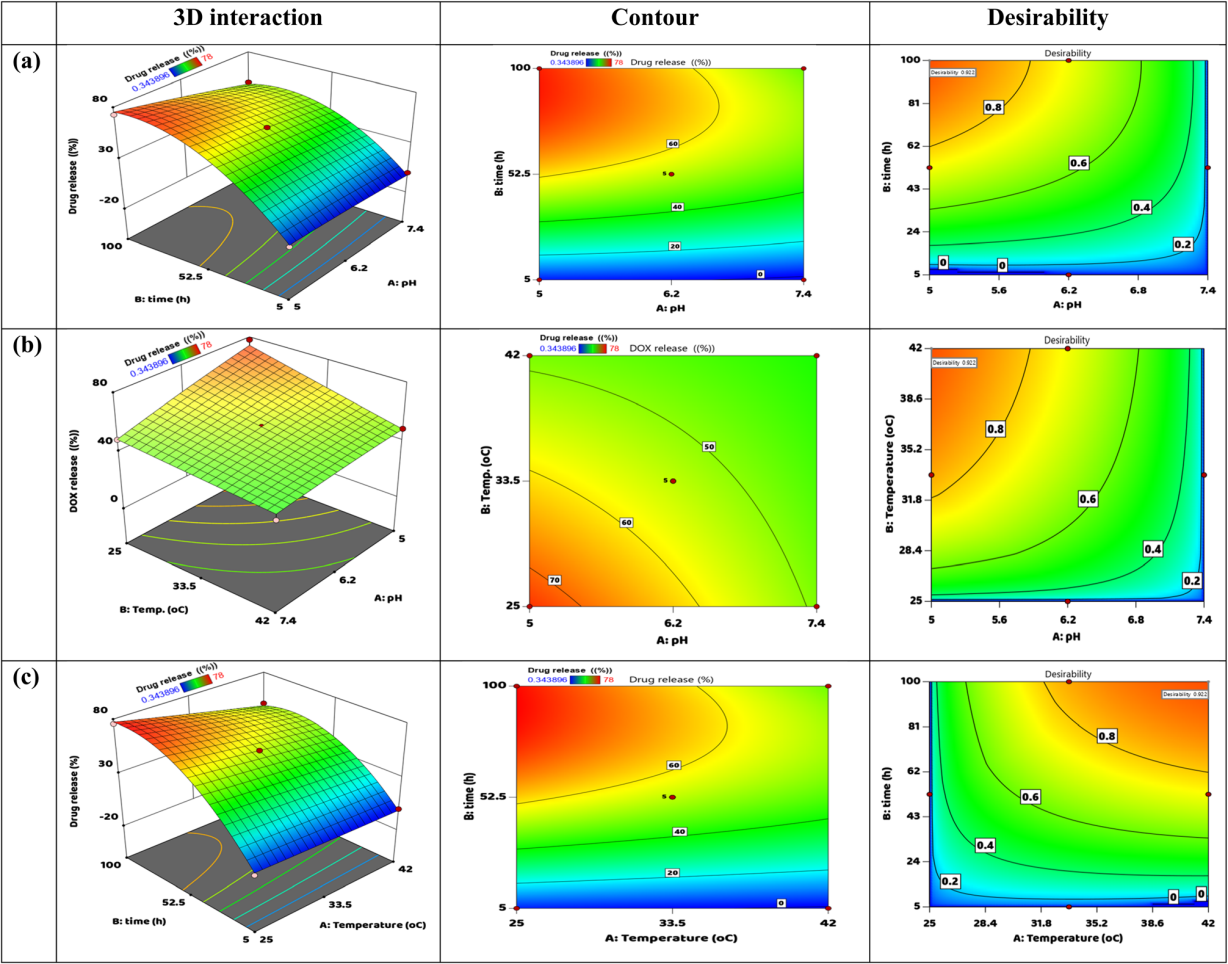
DOX release from the DOX@La-MOF membrane: contour and three-dimensional interaction (a) pH and time interaction, (b) pH and temperature interaction, and (c) time and temperature.

Fig. 9(a) provides a detailed examination of how pH (*A*) and release time (*B*) interact to influence the drug release performance of DOX from the membrane. Three-dimensional surface plots, contour plots, and attractiveness maps are used to demonstrate this. The 3D interaction plot indicates a significant curvature of the surface, suggesting that the release of DOX is notably higher with increased duration and lower pH levels. Specifically, the peak release rate of approximately 78% occurs at 100 hours and pH 5, implying that extended exposure in acidic conditions significantly enhances the release mechanism, potentially due to greater degradation of the MOF matrix or enhanced solubility of DOX. In contrast, minimal or negative release values are noted at higher pH and shorter release times, pointing to ineffective drug diffusion in these scenarios. The contour plot corroborates these findings, as indicated by closely spaced contour lines and a color rise changing from blue (representative low release) to red (indicating high release), which suggests that time is a more dominant factor than pH. The desirability plot quantifies this relationship, pinpointing the optimal conditions for drug release (with desirability near 1.0) occurring at low pH and longer release times. Conversely, desirability sharply declines outside these parameters. Collectively, these plots underscore the importance of both low pH and extended-release durations in maximizing DOX delivery from the membrane, providing vital insights for optimizing formulation conditions.^79^

The combined impact of temperature (*B*) and pH (*A*) on the DOX release from the membrane are shown in Fig. 9(b). A desirability map, a contour graph, and a 3D surface plot are used to depict this data. A smooth, slightly sloping surface is revealed by the 3D surface map, suggesting that temperature and pH have a moderate impact on the drug's release pattern. Increased percentages of DOX release are identified at lower pH levels and moderately elevated temperatures. However, the gentle curvature of the surface implies that the interaction between pH and temperature is not as pronounced as the previously observed interaction involving time and pH. The contour plot reinforces this finding, displaying broad, evenly spaced contours that demonstrate a gradual alteration in drug release as pH and temperature vary. Importantly, drug release remains consistently high and stable at low pH values (approximately 5–6.2), even with moderate changes in temperature, which highlights the stability of DOX release in acidic environments. The attractiveness plot corroborates these results, representative a peak attractiveness score of 0.92 within the low pH range (5–6) and moderate temperature range (∼33–35 °C), which indicates the ideal conditions for optimized drug release. This analysis emphasizes that while temperature has a lesser impact, maintaining acidic pH is crucial for maximizing DOX release, and the interaction between these two variables should be taken into account during formulation optimization efforts.^79^

Using a 3D response surface plot, a 2D contour plot, as well as a desirability map, Fig. 9(c) displays the combined properties of temperature (*A*) and release time (*B*) on the proportion of DOX released from the membrane. The 3D interaction figure shows a clearly curved surface, indicating that temperature and time have a significant synergistic effect on drug release. An increase in the release percentage up to 78% is noted when the release time extends to 100 hours, particularly at lower temperatures in the range of 25–30 °C. In contrast, a significant drop in release percentage occurs at shorter times and elevated temperatures. The contour plot corroborates this finding, illustrated by densely packed, concentric contour lines that shift from blue (signifying low release) to red (representative high release), underlining that longer release times greatly improve DOX release, especially at moderate temperatures. The desirability plot reaffirms this observation, indicating the optimal conditions (attractiveness ∼0.83) in the upper left section of the plot, which corresponds to extended release durations and moderate temperatures. There is a notable reduction in desirability when both time is shorter and temperatures are higher, indicating less favorable release conditions. Overall, these visual representations underscore that an extended release time is the key variable for enhancing DOX delivery, with lower to moderate temperatures further facilitating effective drug release from the membrane.^79^

#### 3.5.4. The attractiveness of the method and model validation

**Fig. 10:**
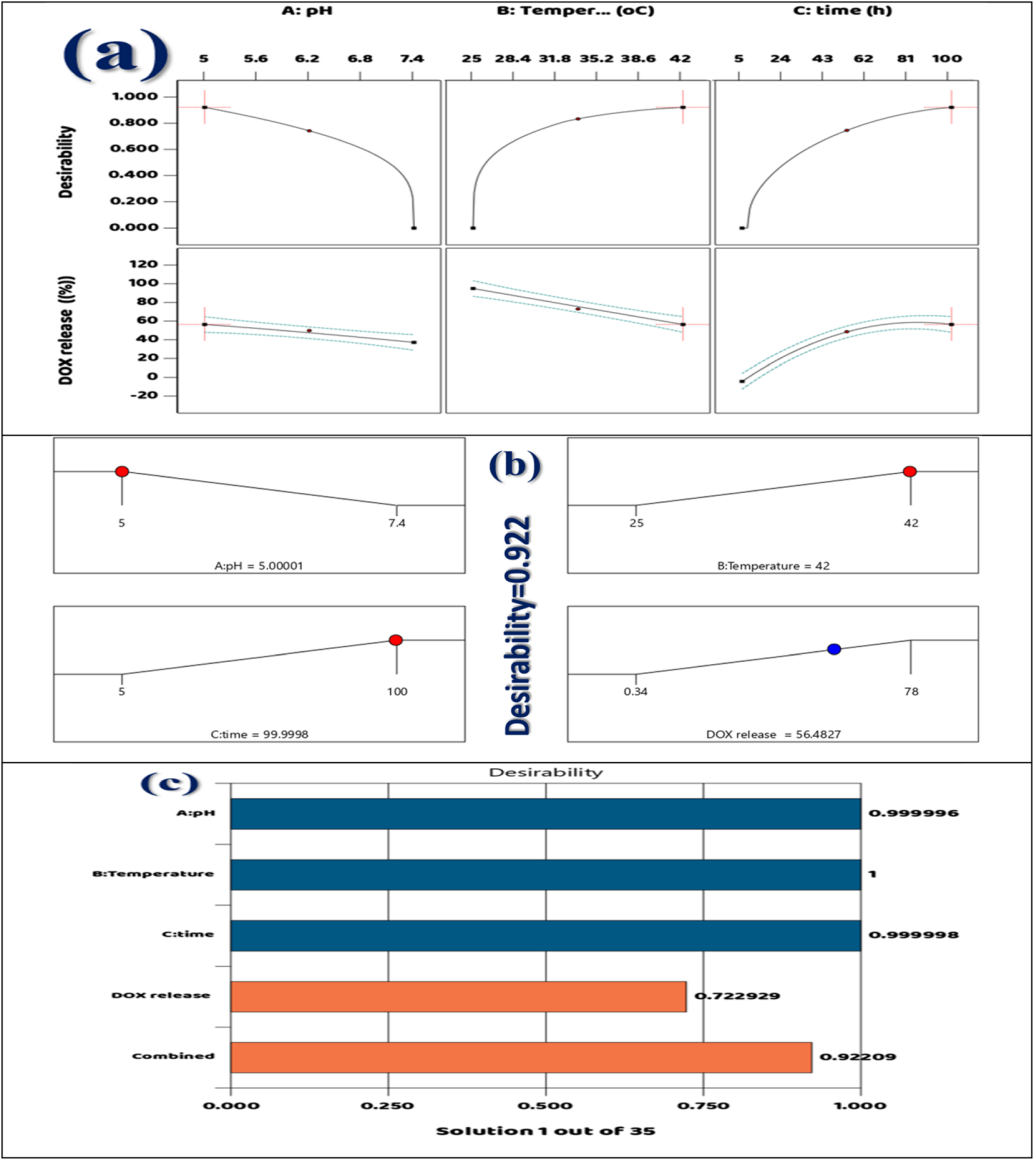
(a) The increasing curiosity about the best numerical answers, (b) desirability of each response, and (c) the display is a bar graph of individual desirability.

Fig. 10(a) illustrates the primary effects of three independent variables pH (*A*), temperature (*B*), and time (*C*) on both the attractiveness (top row) and the percentage of DOX release (bottom row) from the membrane system. The upper panels indicate that the highest desirability arises at a low pH of around 5.0, a high temperature around 42 °C, and an extended duration of about 100 hours, signifying that these situations are ideal for maximizing DOX release. The desirability curve for pH displays a sharp negative slope, indicating a significant decline in release performance as pH transitions since acidic to neutral or alkaline levels. Conversely, the attractiveness curve for temperature rises sharply at higher levels, reflecting a positive effect, albeit less pronounced than that of time.^75^ The variable related to time shows a strong upward trend, reinforcing that extended exposure is the most critical factor for achieving the desired release profile. In the lower row, the actual trends in DOX release align with the desirability plots: DOX release diminishes with increasing pH, experiences a moderate decline with rising temperature, and substantially increases over time. Dotted lines represent confidence intervals, illustrating the statistical significance of all observed trends. In summary, this figure emphasizes that low pH, high temperature, and particularly prolonged release time are essential parameters for optimizing DOX release and facilitating effective drug delivery from the membrane organization.

Fig. 10(b) offerings the outcomes of numerical optimization concerning the DOX release from the membrane system, utilizing the desirability function approach. The graphs illustrate the distinct influences of three independent factors pH (*A*), temperature (*B*), and time (*C*) on the anticipated DOX release, with optimal conditions marked in red or blue. A peak overall desirability of 0.922 is attained under specific settings: pH of 5.00001, temperature of 42 °C, and a time of 99.9998 hours, which correlates with a predicted DOX release rate of 56.4827%. The desirability plots indicate that optimal drug release is enhanced by acidic environments, elevated temperatures, and extended exposure durations. Each line's slope demonstrates the sensitivity of DOX release in response to alterations in each variable, with the most pronounced slope for time, highlighting it as the critical influencing factor. The blue point on the DOX release graph represents the expected performance under these optimized conditions. Collectively, these findings delineate the optimal formulation and operational parameters necessary for enhancing the efficacy of the La-MOF nanofiber drug delivery system.^75^

Fig. 10(c) provides a summary of the desirability rankings for optimizing the DOX release from the membrane, detailing the influence of three parameters: pH (*A*), temperature (*B*), and time (*C*) on the overall outcome. The desirability values for these individual factors are notably high, with pH at 0.999996, temperature at 1.000000, and time at 0.999998. These values propose that the chosen levels for each parameter are nearly ideal for maximizing DOX release. The actual DOX release rate has a desirability of 0.722929, indicating that the achieved percentage of 56.48% closely approaches the targeted goal within the specified optimization constraints. The total combined desirability score of 0.92209 supports the assertion that this particular configuration selected from a total of 35 possible combinations is statistically optimal. This elevated combined score is indicative of the strong correlation of all three independent variables with their ideal conditions, emphasizing that the most effective DOX release occurs under low pH, high temperature, and extended exposure time. Such optimization is expected to enhance the performance and efficiency of the membrane.^75^

### 3.6. Biological procedure

#### 3.6.1. Anti-cancer belongings

##### 3.6.1.1. HepG-2 cell line

**Fig. 11:**
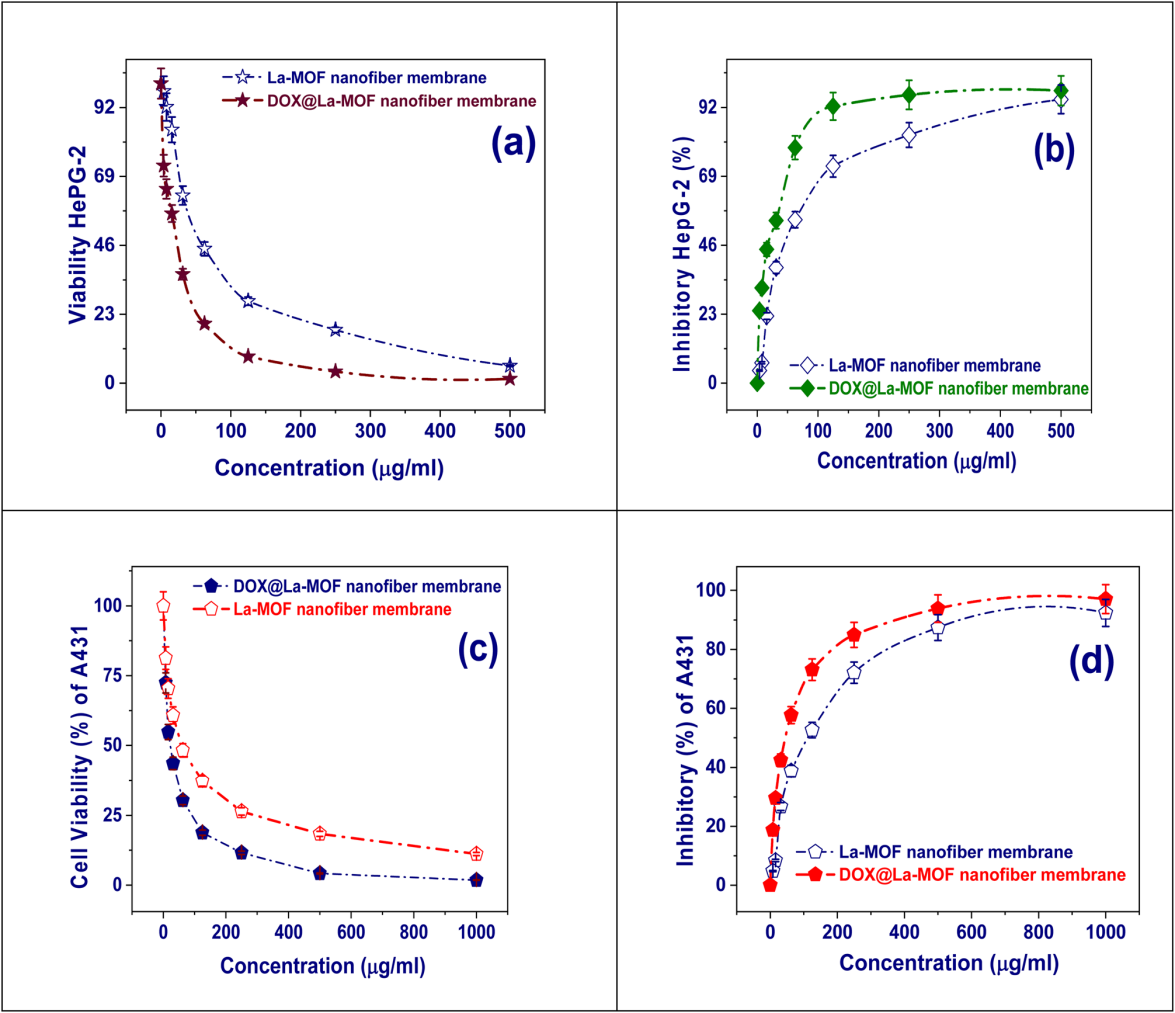
The effectiveness of La-MOF membrane, DOX@ La-MOF membrane, (a) cell feasibility of HePG-2, (b) inhibitory (%) of HePG-2, (c) cell feasibility of A-431, (d) inhibitory (%) of A-431.

The accompanying graphic shows the cytotoxic impacts of the La-MOF membrane and the DOX@La-MOF membrane proceeding HepG-2 liver cancer cells. In particular, the percentages of cell viability and inhibition at different concentrations (0–500 µg mL^−1^) are shown in Fig. 11(a) and (b), respectively. The DOX@La-MOF membrane showed noticeably more cytotoxicity than the La-MOF membrane alone, based on the data, which show a noteworthy dose-dependent correlation. The DOX@La-MOF combination achieved an inhibition rate of over 95% and reduced cell viability to almost 0% at concentrations over 300 µg mL^−1^ (Tables S9 and S10). The membrane, on the other hand, had a less severe effect, subsequent in residual cell feasibility and an inhibition plateau of roughly 85–90% at comparable concentrations.^80^ The addition of DOX, a well-known chemotherapeutic drug, to the membrane matrix is responsible for the observed rise in cytotoxicity. The membrane's versatility as a drug delivery method is one of its main advantages. The biocompatible electrospun mixture of polycaprolactone and chitosan allows for a synergistic, localized, and prolonged release of DOX. HepG-2 cells undergo apoptosis, cellular homeostasis is upset, and reactive oxygen species (ROS) production is increased as a result of these synergistic interactions. In addition to demonstrating improved anticancer activity, the membrane offers a promising alternative for targeted drug administration since it integrates structural stability, biocompatibility, and noticeable therapeutic benefits into a single tiny platform.^80^

##### 3.6.1.2. A-431 cell line

The cytotoxic impacts of La-MOF and DOX@La-MOF membranes on A-431 skin cancer cells are illustrated in the figure. Precisely, Fig. 11(c) measures the percentage of cell viability, and Fig. 11(d) shows the percentage of inhibition over a range of concentrations (0–1000 µg mL^−1^). Both kinds of nanofiber membranes cause a concentration-dependent decrease in A-431 cell viability, according to the data shown in Fig. 11(c). Importantly, the membrane exhibits significantly higher cytotoxicity than the La-MOF membrane alone. The DOX@La-MOF model dramatically reduces cell viability to almost 0% at concentrations over 300 µg mL^−1^. On the other hand, a certain level of cell existence is possible with the La-MOF nanofiber membrane. This result is in line with the data shown in Fig. 11(d), where the DOX@La-MOF nanofiber maintains this level at increasing concentrations and attains over 95% reserve at 300 µg mL^−1^. The La-MOF nanofiber membrane, on the other hand, plateaued at a lower inhibition rate of roughly 85–90%.^81^ Both elements increase oxidative stress levels and encourage cellular death. The electrospun PCL/CS nanofiber matrix is an active vehicle for targeted and continued drug administration, increasing therapeutic belongings and reducing systemic toxicity. Accordingly, the DOX@La-MOF nanofiber system becomes a versatile scaffold with important cytotoxic effects, regulated drug release as well as biocompatibility.^82^ This makes the system a viable option for wound care and tailored cancer treatments in the field of oncology.^35^

#### 3.6.2. Antioxidant activities

**Fig. 12:**
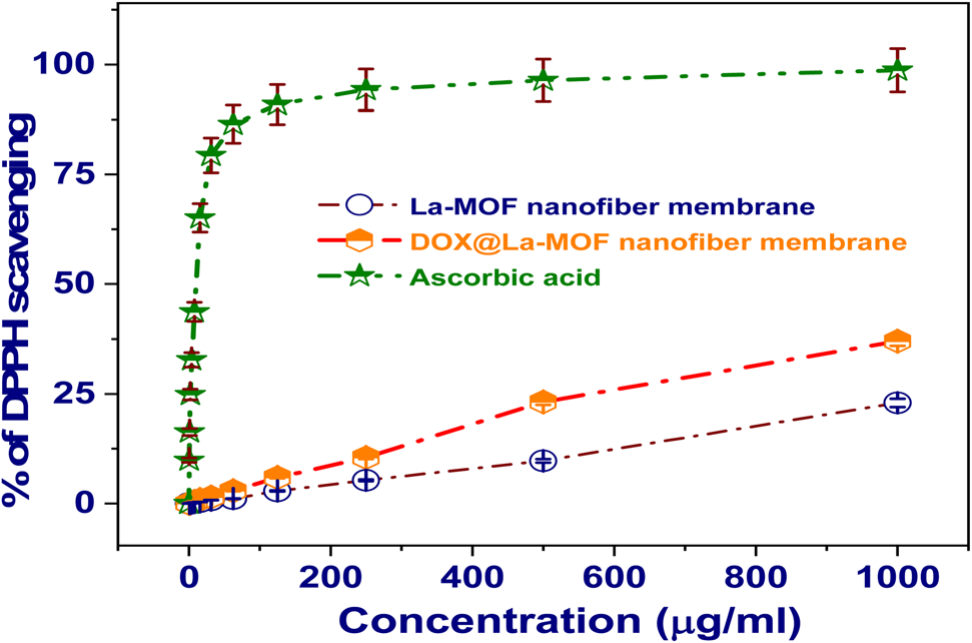
Scavenging of DPPH radicals by La-MOF and DOX@La-MOF membranes.

Fig. 12 demonstrates the DPPH radical scavenging activity for the La-MOF membrane, the DOX@La-MOF membrane, and ascorbic acid, within a concentration gradient of 0–1000 µg mL^−1^. Ascorbic acid functions as the control antioxidant, demonstrating a swift and marked enhancement in scavenging efficiency, surpassing 90% at concentrations under 100 µg mL^−1^ and sustaining nearly total scavenging at elevated concentrations. This behavior underscores its robust capacity for neutralizing free radicals. The La-MOF nanofiber membrane exhibits a notably low level of antioxidant activity, as indicated by DPPH scavenging that incrementally rises to roughly 15% at the maximum concentration. This finding implies a restricted capacity for radical quenching, which can be credited to the lack of useful groups that possess the ability to donate hydrogen atoms. On the other hand, the DOX@La-MOF nanofiber displays a significant enhancement in antioxidant efficacy, recording an approximate scavenging rate of 30% at a concentration of 1000 µg mL^−1^. The observed enhancement in antioxidant activity can be linked to the integration of DOX, which introduces supplementary electron-donating functional groups, including hydroxyl or amine functionalities. This addition likely promotes more efficient interactions with DPPH radicals. The data illustrated in the figure underscores the significant improvement in antioxidant capacity resulting from the loading of DOX onto the La-MOF nanofiber structure. This finding accentuates the efficacy of functional modification in the development of MOF-based systems aimed at augmenting bioactivity.^81^

#### 3.6.3. Antibacterial activities

**Fig. 13:**
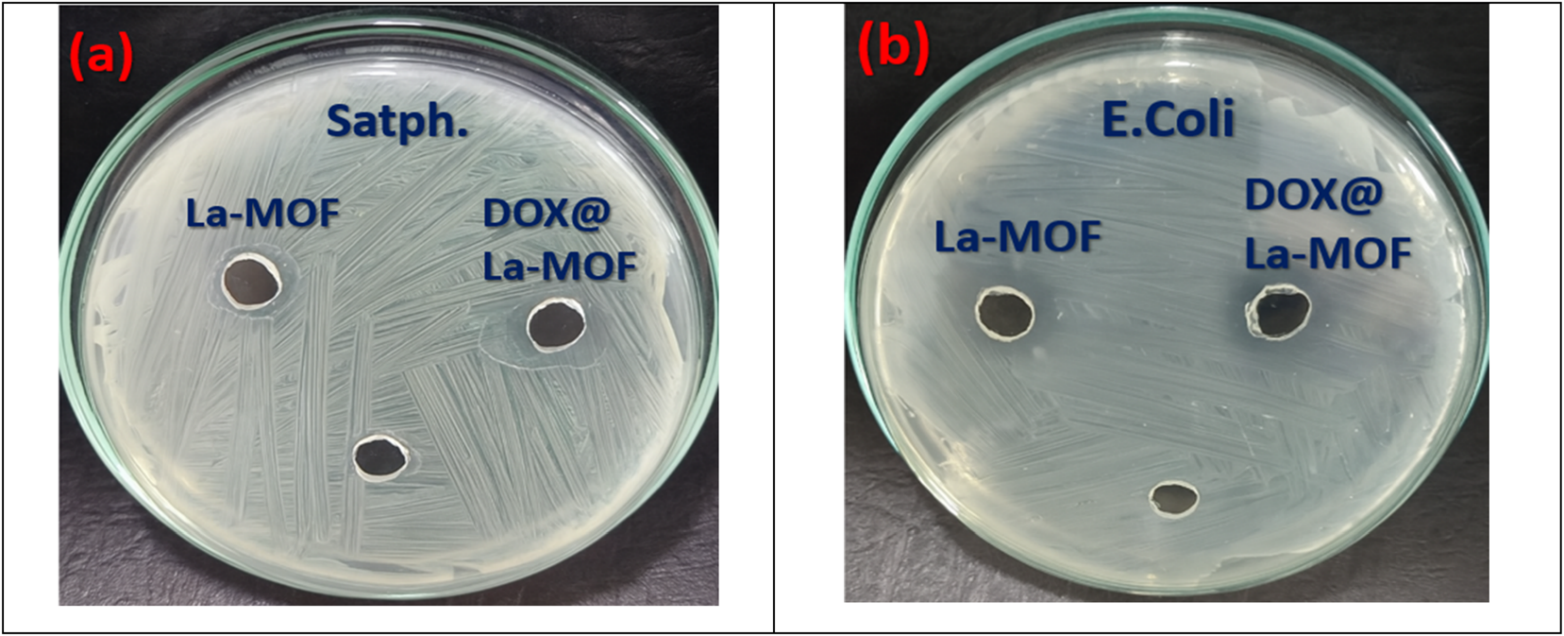
Inhibition zones for La-MOF, and DOX@La-MOF membranes towards: (a) *Staphylococcus aureus*, and (b) *E. coli*.

By incorporating La-MOF into an electrospun substrate made of chitosan as well as polycaprolactone, the antibacterial effectiveness of La-MOF nanofibers and DOX@La-MOF membranes was examined. As shown in Fig. 13, this assessment tested the membranes towards two bacterial strains: the Gram-negative (*Escherichia coli*) as well as the Gram-positive (*Staphylococcus aureus*), using the agar well diffusion method. Notably, Fig. 13(a) demonstrates the presence of inhibition zones for both La-MOF and DOX@La-MOF membranes when challenged with *S. aureus*, where the DOX-loaded membrane exhibits a marginally larger clear zone, suggesting enhanced antibacterial properties. In a similar vein, Fig. 13(b) illustrates the inhibition zones against *E. coli*, further confirming the superior antibacterial efficacy of the DOX@La-MOF membranes sample compared to the unmodified La-MOF. The quantitative information shown in Table 5, that provides the inhibition zone sizes in millimeters, supports these findings. The La-MOF membranes displayed moderate antibacterial activity, registering inhibition zones of 11 mm compared to *S. aureus* as well as 12 mm against *E. coli*. However, the introduction of DOX resulted in an increase in these measurements, yielding inhibition zones of 12 mm for *S. aureus* and 13 mm for *E. coli*. This indicates that DOX significantly enhances the antibacterial properties of the La-MOF. In a comparative analysis, gentamycin, employed as a positive control, demonstrated notably larger inhibition zones of 24 mm for *S. aureus* and 30 mm for *E. coli*, underscoring its potent antimicrobial properties. However, the observed improvements following DOX loading indicate a possible synergistic relationship between the La-MOF matrix and DOX. This interaction is likely attributed to DOX's capacity to interfere with bacterial DNA replication and compromise cell wall integrity. Consequently, these findings support the viability of DOX@La-MOF membranes as effective candidates for antimicrobial applications, particularly in contexts of mild to moderate bacterial resistance.^83^

**Table 5:** La-MOF membrane and DOX@La-MOF membrane have antimicrobial belongings in contrast to Gram-positive (*Staphylococcus aureus*) and Gram-negative (*Escherichia coli*)

Sample	Gram positive bacteria	Gram negative bacteria
	*Staphylococcus aureus* (RCMB 010010) ATCC 25923	*Escherichia coli* (RCMB 010052) ATCC 25955
La-MOF	11	12
DOX@La-MOF	12	13
Regulator (gentamycin)	24	30

## 4. Conclusion

This research successfully introduced a versatile drug delivery organization that replies to changes in pH and temperature. The system involved the encapsulation of doxorubicin (DOX) within a lanthanum-based metal–organic framework (La-MOF) and its incorporation into a biocompatible electrospun chitosan/polycaprolactone nanofiber membrane. A series of extensive physicochemical analyses, including FTIR, XRD, SEM, XPS, and BET, validated the effective synthesis, structural stability, and uniform distribution of La-MOF throughout the fibrous matrix. The DOX@La-MOF nanofiber membrane exhibited significant drug loading efficiency, with a release profile that was notably amplified in acidic pH and elevated temperature conditions, indicating its potential application in targeted cancer treatment. Kinetic analysis indicated that the release of DOX was primarily governed by first-order and Higuchi models, with the mechanism determined by a mixture of diffusion and erosion of the matrix. Statistical optimization by means of Box–Behnken design indicated that pH, duration, and temperature were critical factors affecting drug release, particularly highlighting time as the most influential variable. Additionally, *in vitro* biological assessments demonstrated that this system displayed strong anticancer efficacy against MCF-7, HepG-2, and A-431 cell lines, as well as significant antioxidant and antimicrobial properties. Overall, the DOX@La-MOF membrane represents a highly promising platform for controlled drug delivery that responds to stimuli, with wide-ranging applications in biomedicine, especially in cancer therapy. The combination of MOF technology with biodegradable polymers and innovative design approaches emphasizes the potential of these systems in the evolution of advanced drug delivery methods.

## Author contributions

Fahad T. Alotaibi: conceptualization, data curation, investigation, resources, validation, visualization, writing-review and editing. Malak A. Alamri: conceptualization, data curation, investigation, validation, visualization, writing-review and editing. Lina M. Alneghery: conceptualization, data curation, investigation, validation, visualization, writing-review and editing. Ali M. Alaseem: investigation, validation, visualization, writing-review and editing. Mohamed G. El-Desouky: conceptualization, data curation, investigation, methodology, software, validation, visualization, writing-original draft. Ashraf A. El-Bindary: conceptualization, data curation, investigation, resources, validation, visualization, writing-original draft, writing-review and editing.

## Conflicts of interest

There are no conflicts of interest to declare.

## Supplementary Material

RA-015-D5RA07539D-s001

## Data Availability

The data that support the findings of this study are available from the corresponding author upon reasonable request. Supplementary information (SI) is available. See DOI: https://doi.org/10.1039/d5ra07539d.
