# Supplementary material for: Doxorubicin drug delivery using an electrospun nanofiber membrane of chitosan–polycaprolactone with metal–organic framework: Box–Behnken optimization, anticancer treatment, and antimicrobial activity
Source: RSC Adv. 2025 Dec 10;15(57):49243–68. doi: 10.1039/d5ra07539d (PMC12691353; doi:10.1039/d5ra07539d)
Supplement: RA-015-D5RA07539D-s001 [file RA-015-D5RA07539D-s001.pdf]

Supplementary Information (SI) for RSC Advances.
This journal is © The Royal Society of Chemistry 2025

Doxorubicin drug delivery using an electrospun nanofiber membrane of chitosan–polycaprolactone with metal-organic framework: Box-Behnken optimization, anticancer treatment, and antimicrobial activity

Table S1. Chemical name, formula, and company.

Chemical name	Formula	Company
Chitosan	$C_{56}H_{103}N_9O_3$	Sigma-Aldrich, Germany
Polycaprolactone	$(C_6H_{10}O_2)_n$	Sigma-Aldrich, Germany
Lanthanum nitrate hexahydrate	$La(NO_3)_3.6H_2O$	Sigma-Aldrich, Germany
benzene-1,3,5-tricarboxylic acid	$C_6H_3(CO_2H)_3$	Sigma-Aldrich, Germany
Methanol	CH_3OH	LOBA CHEMIE PVT.LTD, India
Ethanol	C_2H_6O	Sigma-Aldrich, Germany
Sodium hydroxide (99%, AR)	NaOH	Chimmed, Russia
Hydrochloric acid (37%, AR)	HCl	LOBA CHEMIE PVT.LTD, India

Table S2. Instruments and equibments.

Test name	Abbrevation	Instrument name	Company	Illustration
Fourier transformer infrared	FT-IR	A Nicolet IS10 Fourier transform infrared (FTIR) spectrometer	Thermo Fisher Scientific, Waltham, MA, USA	equipped with an attenuated total reflectance accessory and which ran in the 4000-400 cm^{-1} range was used to gather FTIR spectra
Powered X-ray diffraction	PXRD	Siemens diffractometer (model D500, Germany)	Germany	patterns were captured from powder samples through the use of a Siemens diffractometer (model D500, Germany) that was fitted with a Cu-K radiation source (wavelength 1.54 Angstroms (Å)) operating at 30 kV and 20 mA.
Scanning Electron Microscope	SEM	(JSM-6510LV, JEOL Ltd., Tokyo, Japan)	JEOL Ltd., Tokyo, Japan	The morphology of the investigated sorbents was analyzed with the use of a scanning electron microscope
X-ray photoelectron spectroscopy	XPS	K-ALPHA (Themo Fisher Scientific, USA)	Themo Fisher Scientific, USA	Used for determination the elemental analysis for the compound
Braunnar Emmet Teller	BET	Quantachrome Instruments, Anton Paar Quanta Tec, Inc., Boynton Beach, FL,	Quanta Tec, Inc., Boynton Beach, FL, USA	was utilised for surface and pore analysis (Brunauer Emmett-Teller (BET) surface area, porous volume, and pore size), and NovaWin Software (v11.0) was used for data interpretation.

		USA		The BET surface area of material adsorbents was obtained by the application of nitrogen adsorption-desorption isotherms at 77K through the use of a specific analyser (Quadrasorb–EVO, Quantachrome, USA).
UV-visible spectrophotometer	UV spectrophotometer	Perkin-Elmer AA800 spectrophotometer Double beam, with 1 cm cell length.		Measuring the concentration of the adsorbate soulution via using Bear-Lambert law
Energy Dispersive X-ray	EDX	Leo1430VP microscope	Carl Zeiss AG, Jena, Germany	Elemntal analysis of the material
pH meter	pH	HANNA (model 211)	USA	Measuring the acidity or basicity of the solution
Sonication	Ultrasonic	Elmasonic P300H ultrasonic bath, continuous mode, power 380 W	Elma Schmidbauer GmbH, Singen, Germany	Sonication of the material as well as used ton disperse material on the solution as it decrease the particle size of the material
Water bath	Shaking	GFL Orbital Shaker 3017		

Table S3. Summary of DPPH Antioxidant Activity Evaluation.

Parameter	Description
Location of Analysis	Regional Center for Mycology and Biotechnology (RCMB), Al-Azhar University, Egypt.
Assay Type	DPPH (2,2-diphenyl-1-picrylhydrazyl) free radical scavenging assay.
Replicates	Performed in triplicate and averaged.
DPPH Solution	0.004% (w/v) DPPH in methanol; stored at 10°C in the dark.
Sample Preparation	Test compound dissolved in methanol; 40 µL of this solution added to 3 mL DPPH solution.
Reference Compound	Ascorbic acid.
Instrument Used	UV–Visible spectrophotometer (Milton Roy, Spectronic 1201).
Measurement Wavelength	515 nm.
Measurement Time	Absorbance recorded every 1 min for 16 min until stable.
Control	Absorbance of DPPH without antioxidant.
% Inhibition Formula	PI = {[(AC – AT) / AC] × 100} where AC = control absorbance at t=0, AT = sample absorbance at t=16 min.
IC_{50} Determination	Calculated from plotted dose-response curve.

Table S4. Cytotoxicity evaluation for MCF-7 and HepG-2.

Parameter	Description
Location of Analysis	Regional Center for Mycology and Biotechnology (RCMB), Al-Azhar University, Egypt.
Cell Lines Used	MCF-7 (human breast carcinoma), HepG-2 (human liver carcinoma).
Source	American Type Culture Collection (ATCC, Rockville, MD)
Chemicals & Reagents	DMSO, MTT, Trypan blue (Sigma, USA); RPMI-1640, HEPES buffer, L-glutamine, gentamycin, 0.25% Trypsin-EDTA, Fetal Bovine Serum (Lonza, Belgium).
Culture Medium	RPMI-1640 medium supplemented with 10% inactivated fetal calf serum and 50 µg/mL gentamycin.
Incubation Conditions	Maintained at 37°C in a humidified atmosphere with 5% CO_2; subcultured 2–3 times per week.
Plating Density	5×10^4 cells per well in 96-well plates.
Treatment Setup	Tested compounds added in 8 concentrations; each concentration in triplicates.
Controls	Six vehicle controls per plate using media or 0.5% DMSO.
Viability Assay	Cell viability determined using MTT assay after 24 hours incubation.
MTT Procedure	Replace media with 100 µL RPMI-1640 (no phenol red) + 10 µL MTT (12 mM); incubate 4 h at 37°C/5% CO_2.
Solubilization	Remove 85 µL media, add 50 µL DMSO, mix, and incubate 10 min at

Step	37°C.
Measurement	Optical density measured at 590 nm using a SunRise TECAN microplate reader.
Cell Viability Calculation	Viability (%) = (OD_t / ODc) × 100
Data Interpretation	Dose–response plotted and IC_{50} calculated using GraphPad Prism software.

Table S5. Cytotoxicity Evaluation Table for A-431.

**Parameter**	**Description**
Location of Analysis	Regional Center for Mycology and Biotechnology (RCMB), Al-Azhar University, Egypt.
Cell Line Used	A-431 cells (human skin carcinoma cell line).
Source	American Type Culture Collection (ATCC, Rockville, MD).
Chemicals & Reagents	DMSO, MTT, trypan blue (Sigma, USA); RPMI-1640, HEPES buffer, L-glutamine, gentamycin, 0.25% Trypsin-EDTA, Fetal Bovine Serum (Lonza, Belgium).
Culture Medium	RPMI-1640 supplemented with 10% inactivated fetal calf serum and 50 µg/mL gentamycin.
Incubation Conditions	Maintained at 37°C in a humidified 5% CO_2 atmosphere; subcultured 2–3 times/week.
Sample Preparation	Nanofiber sample (10 mg) soaked in 1 mL RPMI-1640 medium for 24 h before use.
Plating Density	5 × 10^4 cells/well in 96-well plates; incubated 24 h before treatment.
Treatment Setup	Tested compounds added in 8 concentrations; each in triplicate.
Controls	Six vehicle controls per plate using media or 0.5% DMSO .
Incubation Period Before MTT	48 hours after compound addition.
MTT Procedure	Replace media with 100 µL RPMI-1640 (no phenol red) + 10 µL MTT (12 mM); incubate 4 h at 37°C/5% CO_2.
Solubilization Step	Remove 85 µL media; add 50 µL DMSO; mix and incubate 10 min at 37°C.
Measurement	Optical density measured at 590 nm using SunRise TECAN microplate reader.
Viability Calculation	% viability = (OD_t / ODc) × 100
Data Interpretation	Plot survival curve; IC_{50} calculated using GraphPad Prism software.

Table S6. True variables, codes, and their BBD levels.

Code	Variables	-1	0	+1
**A**	pH	5	6.2	7.4
**B**	Temperature (^{o}C)	25	33.5	42
**C**	Time (h.)	5	52.5	100

Table S7. Equations used in this work to fit the data of adsorption experiments.

Serial	Equation	Nmae	Description	Ref.
1	$Q_o^{1/3} - Q_t^{1/3} = K_{HC}.t$	Hixson–Crowell model	Q_0 = Initial amount of drug<br>Q_t = Remaining amount of drug at time t<br>K_{HC} = Hixson–Crowell dissolution rate constant<br>t = Time	[1]
2	$Q_t = Q_0 + K_0.t$	Zero-Order	Q_t = Amount of drug released at time ttt<br>Q_0 = Initial amount of drug in the solution (often 0)<br>K_0 = Zero-order release constant (units: concentration/time)<br>t = Time	[2]
3	$\ln Q_t = \ln Q_0 - K_1 \cdot t$	First order	Q_0 = Initial amount of drug<br>Q_t = Amount of drug remaining at time ttt<br>K_1 = First-order rate constant (1/time)<br>t = Time	[3]
4	$\frac{M_t}{M_\infty} = K.t^n$	Korsmeyer–Peppas	Mt = Amount of drug released at time ttt<br>M∞ = Total amount of drug released at infinite time (i.e., final amount)<br>$\frac{M_t}{M_\infty}$ = Fraction of drug released at time t<br>K = Kinetic constant incorporating structural and geometric characteristics<br>n = Release exponent that indicates the mechanism of drug release	[4]
5	$Q_t = K_H\sqrt{t}$	Higuchi	Q_t = Cumulative amount of drug released at time t<br>K_H=Higuchi dissolution constant (units: amount/time^{½})<br>t = Time	[5]

Table S8. The parameter of the kinetic models of DOX release from La-MOF nanofiber membrane

Kinetic model	Value of parameters	
Zero-order	K_o (h)	1.39
	Reduced Chi-Sqr	675.61915
	Residual Sum of Squares	0.95326
	R-Square (COD)	0.9087
	R^2	0.90363
First-order	K_F (h^{-1})	0.4
	Reduced Chi-Sqr	1.40883
	Residual Sum of Squares	0.97737
	R-Square (COD)	0.95526
	R^2	0.95277
Hexson-crowell	K_{HC} (h^{-1})	0.289
	Reduced Chi-Sqr	231.45299
	Residual Sum of Squares	0.92608
	R-Square (COD)	0.85763
	R^2	0.84972
Kosmeyer-peppas	K_F (h^{-1})	0.23
	n	0.00915
	Reduced Chi-Sqr	0.2313
	Residual Sum of Squares	0.92608
	R-Square (COD)	0.85763
	R^2	0.84972
Higuchi	K_H	1.74
	Reduced Chi-Sqr	2083.07687
	Residual Sum of Squares	0.92608
	R-Square (COD)	0.85763
	R^2	0.84972

Table S9. Comparison of different nanocarriers based on MOFs for DOX delivery.

Materials	Drug release	Cell lines	Cell viability	Concentration	Treatment	Ref
LDH-Fe3O4/Cu MOF-DOX-CS@CAR<br>CS (chitosan), CAR (carrageenan hydrogel)	pH 5.5 / 72h / 60%<br>pH 7.4 / 72h / 23%	L929<br>MCF-7	95%<br>50%	62.5 µg/mL	48h	[6]
DOX- CS/Fe_3O_4/Cu-MOF<br>CS (chitosan)	pH 4.5 / 96h / 60%<br>pH 7.4 / 96h / 20%	MCF-7	65%	16 µg/mL	48h	[7]
CS/DOX@Ti-MOF<br>Cs (chitosan)	pH 6.5 / 48h / 76%<br>pH 7.4 / 48h / 10%	MNNG/HOS<br>MDA-MB-231	30%<br>20%	6 µg/mL	48h	[8]
SiO2@Fe3O4-HA-MIL-100-GQDs-DOX<br>HA (hydroxyapatite), GQDs (graphene quantu0m dots)	pH 5 / 70h / 67%<br>pH 7.4 / 70h / 29%	MCF-7	5%	32 µg/mL	72h	[9]
Alg-DOX-Cu MOF-LDH<br>Alg (alginate)	pH 5 / 72h / 69 %<br>pH 6.8 / 72h / 39%<br>pH 7.4 / 72h / 29%	L929<br>MCF-7	90%<br>10%	60 µg/mL	48h	[10]
UiO-66 @P @ DOX<br>P (porphyrin)	pH 4.5/ 200h /90%<br>pH 5.5 / 200h /70%<br>pH 7.4 / 200h /85%	HEK-293<br>HT-29<br>MCF-7<br>MCF-10A	40%<br>60%<br>20%<br>60%	50 µg/mL	48h	[11]

UiO-66 @P @ DOX@RO<br>P (porphyrin), RO (*Rosmarinus officinalis)*	pH 4.5 / 200h / 40%<br>pH 5.5 / 200h / 60%<br>pH 7.4 / 200h / 50%	HEK-293<br>HT-29<br>MCF-7<br>MCF-10A	80%<br>80%<br>65%<br>80%	50 µg/mL	48h	[11]
A520@L@DOX	pH 4.5/ 200h / 94%<br>pH 5.5 / 200h / 97%<br>pH 7.4 / 200h / 96%	HEK-293<br>HeLa<br>MCF-7<br>PC12	95%<br>65%<br>76%<br>70%	50 µg/mL	48h	[12]
A520@L@DOX@L	pH 4.5 / 150h / 36%<br>pH 5.5 / 150h / 49%<br>pH 7.4 / 150h / 88%	HEK-293<br>HeLa<br>MCF-7<br>PC12	96%<br>90%<br>90%<br>83%	50 µg/mL	48h	[12]
DOX@La-MOF nanofiber membrane	pH 5 / 10h / 94.9%<br>pH 6.2 / 100h / 78.8%<br>pH 7.4 / 100h / 53.48%	HepG-2<br>A431	95.2<br>98.4	94.6 µg/mL	50	This study

Table S10. Using different MOFs with different coating agents on different cell lines.

MOFs	Coating agents	Cell lines	Ref
Silver-Based MOF	Chitosan	L929	[13]
BioMOF	Chitosan	HUVEC	[14]
UiO-66	Fe_3O_4 Nanoparticles	HeLa, NIH/3T3	[15]
UiO-66	Aloe vera Biopolymer	HFFF2	[16]
UiO-66	PEG	MCF-7	[17]
UiO-68	Aptamer	MDA-MB-23 , MCF-10A	[18]

Cu-MOF	L-lysine	MCF-7 , MCF-10A	[19]
Cu-MOF	Aptamer	Aptamer	[20]
MIL-100(Fe)	Silica	MCF-7 , MCF-10A	[21]
MIL-100(Fe)	PEG	MCF-7	[22]
ZIF-8	Chitosan & Folic acid	MCF-7	[23]
MIL-88B	Chitosan & Folic acid	M109	[24]
Ni/Ta-MOF	Chitosan & Folic acid	MCF-7 , HepG2	[25]
Zn-N-MOF	Chitosan & Folic acid	HCT116	[26]
MOF-5	Chitosan & Alginate	HEK-293 , PC12 , HepG2	[27]
MOF-5	Carboxymethylcellulose, Aptamer	HeLa , 4TA	[28]
UiO-66-NH2	Porphyrin	MCF-7 , HT-29	[11]
beta- CD-MOF	Glutamine	MCF-7 , AGS	[29]
Bio-MOF-11	Pectin Biopolymer	SW489	[30]
Fe-BTC MOF	Liposome	MCF-7	[31]
A520	Tp Extract	MCF-7 , HeLa, HEK-293, PC12	[12]
DOX@La-MOF nanofiber membrane	Chitosan and polycprolactone	HepG-2, A431	This study

References

[1] K. Ramteke, P. Dighe, A. Kharat, S. Patil, Mathematical models of drug dissolution: A review, Sch. Acad. J. Pharm, 3 (2014) 388-396.
[2] B. Narasimhan, R. Langer, Zero-order release of micro-and macromolecules from polymeric devices: the role of the burst effect, Journal of controlled release, 47 (1997) 13-20.
[3] N. Mulye, S. Turco, A simple model based on first order kinetics to explain release of highly water soluble drugs from porous dicalcium phosphate dihydrate matrices, Drug development and industrial pharmacy, 21 (1995) 943-953.
[4] A. Talevi, M.E. Ruiz, Korsmeyer-Peppas, Peppas-Sahlin, and Brazel-Peppas: Models of drug release, The ADME Encyclopedia: A Comprehensive Guide on Biopharmacy and Pharmacokinetics, Springer2022, pp. 613-621.
[5] D. Paul, Elaborations on the Higuchi model for drug delivery, International journal of pharmaceutics, 418 (2011) 13-17.
[6] A. Taghikhani, M. Babazadeh, S. Davaran, E. Ghasemi, Facile preparation of a pH-sensitive biocompatible nanocarrier based on magnetic layered double hydroxides/Cu MOFs-chitosan crosslinked κ-carrageenan for controlled doxorubicin delivery to breast cancer cells, Colloids and Surfaces B: Biointerfaces, 243 (2024) 114122.

[7] M. Abbasian, M. Khayyatalimohammadi, In-situ forming Cu-based metal-organic framework in the presence of chitosan-Fe3O4 nanohybrids: A pH-sensitive carrier for controlled release of doxorubicin, International Journal of Biological Macromolecules, 278 (2024) 134224.
[8] Y. Zeng, J. Yuan, Z. Ran, X. Zhan, X. Li, H. Ye, J. Dong, G. Cao, Z. Pan, Y. Bao, Chitosan/NH2-MIL-125 (Ti) scaffold loaded with doxorubicin for postoperative bone tumor clearance and osteogenesis: An in vitro study, International Journal of Biological Macromolecules, 263 (2024) 130368.
[9] S. Karimi, V. Zeyni, H. Namazi, A fluorescent system based on graphene quantum dots-capped magnetic hydroxyapatite-MIL-100 metal-organic frameworks for pH-sensitive and controlled release of DOX, Diamond and Related Materials, 140 (2023) 110502.
[10] S. Karimi, H. Rasuli, R. Mohammadi, Facile preparation of pH-sensitive biocompatible alginate beads havening layered double hydroxide supported metal-organic framework for controlled release from doxorubicin to breast cancer cells, International Journal of Biological Macromolecules, 234 (2023) 123538.
[11] S. Ahmadi, V. Jajarmi, M. Ashrafizadeh, A. Zarrabi, J.T. Haponiuk, M.R. Saeb, E.C. Lima, M. Rabiee, N. Rabiee, Mission impossible for cellular internalization: When porphyrin alliance with UiO-66-NH2 MOF gives the cell lines a ride, Journal of Hazardous Materials, 436 (2022) 129259.
[12] H. Daneshgar, M. Bagherzadeh, S. Sojdeh, M. Safarkhani, M. Edrisi, A. Ojaghi, S. Ahmadi, M. Kiani, N. Rabiee, Discovery of valley-hill structures on the surface of MOFs: Enhancing DOX diffusion and release through nature-made channels, Nano Materials Science, (2024).
[13] S.M. Dehnavi, M. Barjasteh, S.A. Seyedkhani, S.Y. Rahnamaee, R. Bagheri, A novel silver-based metal-organic framework incorporated into nanofibrous chitosan coatings for bone tissue implants, International Journal of Pharmaceutics, 640 (2023) 123047.
[14] R. Abazari, A.R. Mahjoub, F. Ataei, A. Morsali, C.L. Carpenter-Warren, K. Mehdizadeh, A.M. Slawin, Chitosan immobilization on bio-MOF nanostructures: a biocompatible pH-responsive nanocarrier for doxorubicin release on MCF-7 cell lines of human breast cancer, Inorganic Chemistry, 57 (2018) 13364-13379.
[15] H.-X. Zhao, Q. Zou, S.-K. Sun, C. Yu, X. Zhang, R.-J. Li, Y.-Y. Fu, Theranostic metal–organic framework core–shell composites for magnetic resonance imaging and drug delivery, Chemical science, 7 (2016) 5294-5301.
[16] H. Nabipour, S. Rohani, Zirconium metal organic framework/aloe vera carrier loaded with naproxen as a versatile platform for drug delivery, Chemical Papers, 77 (2023) 3461-3470.
[17] V. Gupta, S. Mohiyuddin, A. Sachdev, P. Soni, P. Gopinath, S. Tyagi, PEG functionalized zirconium dicarboxylate MOFs for docetaxel drug delivery in vitro, Journal of Drug Delivery Science and Technology, 52 (2019) 846-855.
[18] W.-H. Chen, X. Yu, A. Cecconello, Y.S. Sohn, R. Nechushtai, I. Willner, Stimuli-responsive nucleic acid-functionalized metal–organic framework nanoparticles using pH-and metal-ion-dependent DNAzymes as locks, Chemical science, 8 (2017) 5769-5780.
[19] M.R. Moghadam, S. Karimi, H. Namazi, A targeted biosystem based on l-lysine coated GO@ rod-Cu (II) metal-organic frameworks for pH-controlled co-delivery of doxorubicin and curcumin, Food Bioscience, 58 (2024) 103578.
[20] M. Falsafi, M. Zahiri, A.S. Saljooghi, K. Abnous, S.M. Taghdisi, A. Sazgarnia, M. Ramezani, M. Alibolandi, Aptamer targeted red blood cell membrane-coated porphyrinic copper-based MOF for guided photochemotherapy against metastatic breast cancer, Microporous and Mesoporous Materials, 325 (2021) 111337.

[21] F. Parsa, M. Setoodehkhah, S.M. Atyabi, Loading and release study of ciprofloxacin from silica-coated magnetite modified by iron-based metal-organic framework (MOF) as a nonocarrier in targeted drug delivery system, Inorganic Chemistry Communications, 155 (2023) 111056.
[22] P. Yadav, S. Kumari, A. Yadav, P. Bhardwaj, M. Maruthi, A. Chakraborty, P. Kanoo, Biocompatible Drug Delivery System Based on a MOF Platform for a Sustained and Controlled Release of the Poorly Soluble Drug Norfloxacin, ACS omega, 8 (2023) 28367-28375.
[23] M. Ghaderpour, S. Kashanian, M. Nazari, M. Motiei, S. Sajadimajd, Targeted Delivery of Letrozole Using a Modified Metal–Organic Framework as a Promising Candidate in Breast Cancer Therapy, BioNanoScience, (2024) 1-14.
[24] S. Dehghani, M. Hosseini, S. Haghgoo, V. Changizi, H. Akbari Javar, M. Khoobi, N. Riahi Alam, Multifunctional MIL-Cur@ FC as a theranostic agent for magnetic resonance imaging and targeting drug delivery: in vitro and in vivo study, Journal of drug targeting, 28 (2020) 668-680.
[25] S.-s. Jalaladdiny, A. Badoei-dalfard, Z. Karami, G. Sargazi, Co-delivery of doxorubicin and curcumin to breast cancer cells by a targeted delivery system based on Ni/Ta core-shell metal-organic framework coated with folic acid-activated chitosan nanoparticles, Journal of the Iranian Chemical Society, 19 (2022) 4287-4298.
[26] Z. Khatibi, N.M. Kazemi, S. Khaleghi, Targeted and biocompatible NMOF as efficient nanocomposite for delivery of methotrexate to colon cancer cells, Journal of Drug Delivery Science and Technology, 73 (2022) 103441.
[27] N. Rabiee, M. Bagherzadeh, M. Jouyandeh, P. Zarrintaj, M.R. Saeb, M. Mozafari, M. Shokouhimehr, R.S. Varma, Natural polymers decorated MOF-MXene nanocarriers for co-delivery of doxorubicin/pCRISPR, ACS applied bio materials, 4 (2021) 5106-5121.
[28] S. Javanbakht, A. Hemmati, H. Namazi, A. Heydari, Carboxymethylcellulose-coated 5-fluorouracil@ MOF-5 nano-hybrid as a bio-nanocomposite carrier for the anticancer oral delivery, International journal of biological macromolecules, 155 (2020) 876-882.
[29] P. Sadeh, S. Zeinali, B. Rastegari, I. Najafipour, Functionalization of β-cyclodextrin metal-organic frameworks with gelatin and glutamine for drug delivery of curcumin to cancerous cells, Heliyon, 10 (2024).
[30] H. Nabipour, Y. Hu, Development of fully bio-based pectin/curcumin@ bio-MOF-11 for colon specific drug delivery, Chemical Papers, 76 (2022) 2969-2979.
[31] A. Karami, A. Ahmed, R. Sabouni, G.A. Husseini, M. Al Sharabati, N. AlSawaftah, V. Paul, Hybrid liposome/metal–organic framework as a promising dual-responsive nanocarriers for anticancer drug delivery, Colloids and Surfaces B: Biointerfaces, 217 (2022) 112599.
